# Diagnosis and management of femoroacetabular impingement syndrome (FAIS): a survey of contemporary physiotherapy practice

**DOI:** 10.1186/s12891-025-08708-7

**Published:** 2025-10-07

**Authors:** Peter R. Lawrenson, Helen P. French, Benita Olivier, Karen L. Barker, Joanne L. Kemp, Jackie L. Whittaker, Stephanie J. Woodley

**Affiliations:** 1 Department of Anatomy, School of Biomedical Sciences, University of Otago, Dunedin, New Zealand; 2 School of Health and Rehabilitation Sciences, The University of Queensland, Brisbane, Queensland Australia; 3 Innovation and Research Centre, Community and Oral Health, Metro North Hospital and Health Service, Brisbane, Australia; 4 La Trobe Sport and Exercise Medicine Research Centre, School of Allied Health Human Services and Sport, La Trobe University, Melbourne, Australia; 5 School of Physiotherapy, Royal College of Surgeons in Ireland, Dublin, Ireland; 6 Centre for Healthy Living Research, Department of Sport, Health Sciences and Social Work, Oxford Institute of Applied Health Research, Oxford Brookes University, Oxford, UK; 7 Wits Cricket Research Hub for Science, Medicine and Rehabilitation, Department of Physiotherapy, School of Therapeutic Sciences, Faculty of Health Sciences, University of the Witwatersrand, Johannesburg, South Africa; 8 Nuffield Department of Orthopaedics, Rheumatology and Musculoskeletal Sciences, University of Oxford, Oxford, UK; 9 Department of Physiotherapy, Faculty of Medicine, Centre for Aging SMART, University of British Columbia, Vancouver, Canada; 10 Arthritis Research Canada, Vancouver, Canada

**Keywords:** Femoroacetabular impingement syndrome, FAIS, Diagnosis, Education, Management, Physiotherapy, Physical therapy

## Abstract

**Background:**

Femoroacetabular impingement syndrome (FAIS) is a motion-related hip disorder characterised by altered hip-joint morphology and symptoms. Recent consensus statements have provided guidance on the diagnosis and management of FAIS but given the knowledge gaps in translating research into practice, it is unclear at what level this is being utilised by primary contact physiotherapists. This study undertook a cross-sectional multi-centre international survey to describe contemporary physiotherapy practice for the diagnosis and management of femoroacetabular impingement syndrome (FAIS).

**Methods:**

An online survey comprising 32 questions based around current consensus recommendations for the diagnosis and management of FAIS, was developed. The survey was distributed to six English-speaking countries (Australia, Canada, Ireland, New Zealand, South Africa and the United Kingdom) where physiotherapists work as primary contact practitioners. Questions were answered with a 5-point Likert scale. To describe the ‘most commonly’ utilised tools for diagnosis and management, the two highest ranked responses (‘always’ and ‘often’) were combined for analysis and presented as a percentage of total respondents.

**Results:**

Four hundred and twenty-nine (72%) of eligible respondents were included. Respondents varied across the six countries, 58% were female, and most worked in private practice (70%). When diagnosing FAIS, patient-reported signs/symptoms (90%), functional tests (88%), special tests (87%), and strength assessments (70%) were ‘most commonly’ used, while imaging (60%) and balance assessment (33%) were less frequently implemented. Most respondents employed strengthening exercises (97%) and education (96%) in their management of FAIS, and some utilised range of motion/stretching (62%), and manual therapy (62%). Half of the respondents (52%) use patient-reported outcome measures to assess treatment effectiveness.

**Conclusions:**

Our findings of physiotherapy diagnosis and management of FAIS from six countries broadly aligns with contemporary expert recommendations. Physiotherapy diagnosis of FAIS in practice is guided by patient-reported symptoms, and functional and special tests. Central to physiotherapy management is exercise and advice/education. Other modalities are less frequently utilised.

**Clinical trial number:**

Not applicable.

**Supplementary Information:**

The online version contains supplementary material available at 10.1186/s12891-025-08708-7.

## Background

Femoroacetabular impingement syndrome (FAIS) is a motion-related hip disorder characterised by the presence of altered hip-joint morphology and symptoms [1]. This altered morphology contributes to premature bony contact of the femoral head/neck junction and acetabular rim [2], which can result in pain, intra-articular pathology, and the potential for the development of hip osteoarthritis (OA) [3]. The symptoms and impairments associated with FAIS impact on quality-of-life and participation in physical activity at a young age [4]. With OA recognised as a growing issue among young people [5], and hip OA identified as a significant contributor to the global burden of disease [6], there is a need to investigate the management of conditions (e.g., FAIS) linked to the pathogenesis of end-stage hip joint degeneration.

Current management options for FAIS broadly consist of conservative management, encompassing education, lifestyle modification and physiotherapy-led rehabilitation; and intrusive interventions, such as surgery [4, 7]. Surgical interventions are most prominently reported due to their proposed impact on symptoms, directly targeting the altered bony morphology associated with FAIS [8]. However, surgery has limitations, including cost, inherent risks, and a lack of longitudinal data to support its effectiveness in preventing joint degeneration [5, 9]. Physiotherapist-led interventions are recognised as an adjunct to surgical management or as a stand-alone non-invasive treatment alternative. Physiotherapy-led management does not alter hip morphology but rather uses exercise therapy, manual therapy, and advice, among other interventions to target specific hip-related impairments. This approach is recognised as a cost-effective and low risk alternative to invasive surgical techniques [4], yet, there is dearth of evidence surrounding the most effective physiotherapist-led approach(es). Patients with FAIS report negative expectations regarding physiotherapy-led treatment [10], highlighting a need to better understand exactly what knowledge, beliefs and interventions are being incorporated within contemporary physiotherapy management.

Recent consensus statements addressing the diagnosis and management of FAIS have highlighted impairments, diagnostic criteria, and avenues for future targeted interventions [1, 11, 12]. Given known gaps in translating research to practice [13] it is unclear at what level this information is being utilised by primary contact clinicians. This study aimed to survey physiotherapists across six countries where physiotherapists are primary contact clinicians to clarify the current practice patterns around the diagnosis and management of FAIS. It is anticipated that an improved awareness of contemporary physiotherapy practice could assist in identifying inconsistencies in treatment priorities within clinical practice, inform educational opportunities, and influence future research, in particular clinical trials testing the effectiveness of physiotherapist-led interventions.

## Methods

This study was conducted in accordance with the Declaration of Helsinki. Ethical approval was provided by the institutional research ethics committee in the country (New Zealand) from which the survey was distributed (University of Otago Human Ethics Committee; Reference Number, D20/175). Before undertaking the survey, participants were provided with information on the project through a hyperlink on the survey landing page. Consent was gained through participants selecting the option to ‘agree’ to take part in the survey after reading the participant information, following which access to the survey was enabled. This observational cross-sectional study followed the Strengthening the Reporting of Observational Studies in Epidemiology (STROBE) Statement [14].

### Study design and recruitment

The online survey was completed anonymously by a convenience sample of registered physiotherapists in six countries: Australia, Canada, Ireland, New Zealand, South Africa and the United Kingdom. Countries where physiotherapists can work as primary contact practitioners (i.e., patients can present to physiotherapy without prior referral from a medical professional) and have English as an official language were selected for inclusion. To be eligible to participate, survey respondents were required to indicate that they were currently registered physiotherapists with their regional or national regulatory body; self-report that they treat patients with hip-related pain as a regular part of their practice and be able to understand written English.

### Survey development and pilot

The online survey was developed specifically for this study using published FAIS recommendations [15, 16], and focused on two domains of interest– diagnosis and management. A literature search identified expert consensus statements addressing classification of FAIS, and the diagnosis and management of hip-related pain [1, 11, 17], which guided the specific question parameters within each domain. The initial survey, modelled on a published practitioner survey [18] consisted of 65 questions and was divided into three sections: (i) practitioner demographic profile; and physiotherapy (ii) diagnosis and (iii) management of FAIS. To assess content validity, the author team comprising researchers and/or clinicians in the field of hip-related pain, reviewed all questions within each domain and provided feedback on clarity, relevance, survey completion time, and flow. The author team further refined the survey through an additional two iterations, with the final version including 32 questions (single and multiple-choice questions and 5-point Likert scales to assess level of agreement) (Online Supplementary Material: Survey Questions). To confirm the interpretability of the final 32-question survey before wider distribution and its utility on different mobile platforms, it was piloted by six independent physiotherapists, one from each country, whose data were not included in the analyses.

### Survey distribution and data collection

The survey was distributed using an anonymous web-based link through Qualtrics^XM^ survey software. Members of musculoskeletal, sports and exercise, and orthopaedic special interest groups in each country’s national physiotherapy organisation were invited directly to participate through email. We estimated 14,000 eligible practitioners across the six countries and using an expected response rate of 7–11% [19, 20] a total target sample of approximately 975–1,535 physiotherapy practitioners was expected. Social media advertising was undertaken to reach eligible physiotherapists who were not members of their professional groups; survey respondents went in the draw to win one of twenty $50 e-vouchers. The survey period ran for 8 weeks and following this the link automatically deactivated. Partially completed surveys, where > 80% of questions had been completed, were automatically included in the analysis [19]. Participant information and consent forms were integrated into the online survey, with all participants required to review these documents and select “agree” before progressing to the survey questions. Informed consent was obtained from all participants who provided data to be included in this study. Raw survey data were collected in Qualtrics^XM^ and exported to Microsoft Excel for analysis.

### Data analyses

Data were analysed descriptively. Mean and standard deviation or median and interquartile range were calculated for numerical data, depending on normality. Frequency counts were calculated for ordinal and nominal data. Mean ranks were determined for frequency responses, using the Likert scale (never, rarely, sometimes, often, and always; 0 = never, 4 = always). To describe the ‘most commonly’ utilised tools for diagnosis and management, responses of ‘often’ and ‘always’ were combined for analysis [21], and presented as a percentage of total respondents.

## Results

### Survey response

A total of 682 physiotherapists agreed to participate in the survey. Eighty-eight respondents did not meet the eligibility criteria (not registered = 14; not practising in one of the participating countries = 74) and were excluded. Of the 594 eligible physiotherapists, 429 (72%) completed ≥ 80% of the questions and were included in the analysis. Physiotherapists practicing in Ireland represented the largest proportion of valid respondents (38%, 152/429) with the lowest proportion from Canada (4%, 19/429) (Table 1).

### Practitioner demographic profile

Over half of respondents were female (58%, 250/429), with most working in private practice (70%, 302/429) and hospital outpatient departments (23%, 99/429). Nearly two-thirds (62%, 264/429) saw patients with FAIS 1–2 times per month, and their physiotherapy experience ranged between > 16 years (37%, 158/429) to < 5 years (18%, 76/429). Most respondents (95%, 406/429) were members of their country’s national physiotherapy association, and of these 75% (305/406) were affiliated with a clinical interest group (Table 1).

**Table 1:** Respondents demographics and profile

Demographics	*n* (%) ^a^						
	Australia (*n* = 89)	Canada (*n* = 35)	Ireland (*n* = 152)	New Zealand (*n* = 55)	South Africa (*n* = 44)	United Kingdom (*n* = 54)	Total (*n* = 429)
Sex							
Male	41 (46.1)	19 (54.3)	52 (34.2)	26 (47.3)	7 (15.9)	31 (57.4)	176 (41.0)
Female	47 (52.8)	16 (45.7)	99 (65.1)	29 (52.7)	36 (81.8)	23 (42.6)	250 (58.3)
Prefer not to answer	1 (1.1)	-	1 (0.7)	-	1 (2.3)	-	3 (0.7)
Practice setting ^b^							
Private practice	72 (80.9)	28 (80.0)	98 (64.5)	48 (87.3)	39 (88.6)	17 (31.5)	302 (70.4)
Academic (education)	10 (11.2)	3 (8.6)	8 (5.3)	1 (1.8)	1 (2.3)	1 (1.9)	24 (5.6)
Specialist centre	0	3 (8.6)	5 (3.3)	1 (1.8)	1 (2.3)	5 (9.3)	15 (3.5)
Hospital inpatient	5 (5.6)	5 (14.3)	10 (6.6)	5 (9.1)	11 (25.0)	3 (5.6)	39 (9.1)
Hospital outpatient	10 (11.2)	3 (8.6)	43 (28.3)	6 (10.9)	7 (15.9)	30 (55.6)	99 (23.1)
Public sector	5 (5.6)	1 (2.9)	32 (21.1)	4 (7.3)	3 (6.8)	3 (5.6)	48 (11.2)
High performance sport	15 (16.9)	5 (14.3)	9 (5.9)	6 (10.9)	7 (15.9)	8 (14.8)	50 (11.7)
Other	8 (9.0)	2 (5.7)	3 (2.0)	0	1 (2.3)	4 (7.4)	18 (4.2)
Clinical physiotherapy experience							
< 5 years	22 (24.7)	3 (8.6)	13 (8.6)	22 (40.0)	8 (18.2)	8 (14.8)	76 (17.7)
5 to 10 years	22 (24.7)	13 (37.1)	29 (19.1)	13 (23.6)	8 (18.2)	15 (27.8)	100 (23.3)
11 to 15 years	20 (22.5)	6 (17.1)	36 (23.7)	6 (10.9)	14 (31.8)	13 (24.1)	95 (22.2)
> 16 years	25 (28.1)	13 (37.1)	74 (48.7)	14 (25.5)	14 (31.8)	18 (33.3)	158 (36.8)
Member of national physiotherapy association							
Yes	79 (88.8)	34 (97.1)	149 (98.0)	48 (87.3)	42 (95.5)	54 (100.0)	406 (94.6)
No	10 (11.2)	1 (2.9)	3 (2.0)	7 (12.7)	2 (4.6)	0	23 (5.4)
Member of clinical interest group ^b^							
Musculoskeletal/manipulative therapy	25 (31.7)	15 (44.1)	90 (60.4)	10 (20.8)	34 (81.0)	18 (33.3)	192 (47.3)
Sports/exercise therapy	45 (57.0)	13 (38.2)	66 (44.3)	13 (27.1)	24 (57.1)	13 (24.1)	174 (42.9)
Orthopaedics	2 (2.5)	15 (44.1)	34 (22.8)	0	21 (50.0)	10 (18.5)	82 (20.2)
Other	5 (6.3)	4 (11.8)	12 (8.1)	5 (10.4)	4 (9.5)	2 (3.7)	32 (7.9)
Not a member	22 (27.9)	5 (14.7)	31 (20.8)	20 (41.7)	0	23 (42.6)	101 (24.9)
Percentage clinical practice relating to hip pain, median (interquartile range) ^a^	22 (19.0)	29 (17.5)	20 (18.3)	15 (13.5)	25.5 (20.0)	30 (20.0)	20 (17.0)
Number of patients with FAIS seen per month							
1 to 2 patients	49 (55.1)	18 (51.4)	109 (71.7)	35 (63.6)	29 (65.9)	24 (44.4)	264 (61.5)
3 to 5 patients	20 (22.5)	12 (34.3)	36 (23.7)	18 (32.7)	13 (29.5)	17 (31.5)	116 (27.0)
6 to 10 patients	13 (13.5)	2 (5.7)	7 (4.6)	1 (1.8)	2 (4.5)	3 (5.6)	27 (6.3)
> 10 patients	8 (9.0)	3 (8.6)	0	1 (1.8)	0	10 (18.5)	22 (5.1)

^a^ Except where indicated. ^b^ Percentage sums to greater than 100% as participants were able to provide more than one response.

### Diagnosis of FAIS

**Fig. 1:**
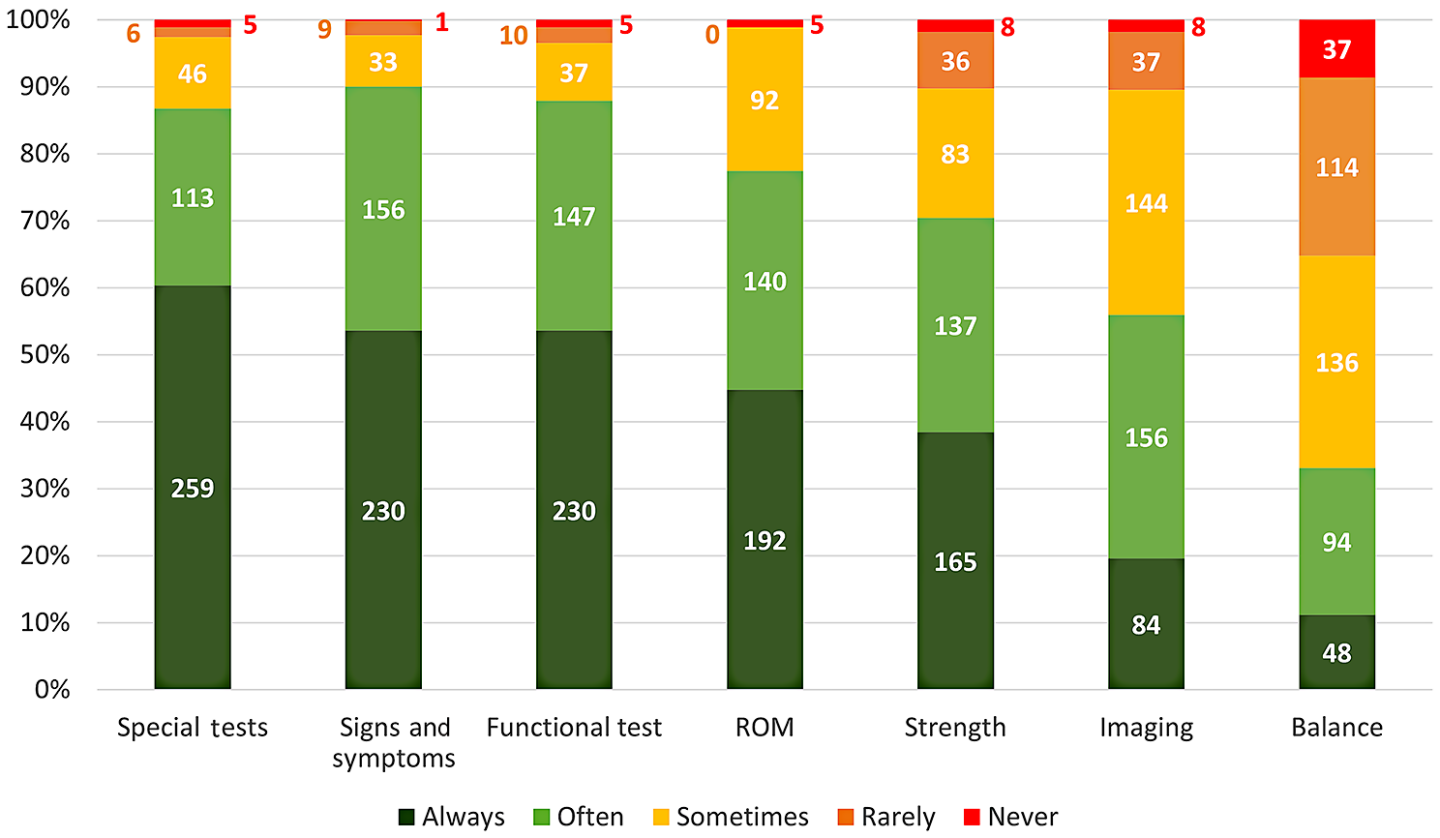
The frequency of assessment tools utilised in the diagnosis of FAIS

Respondents utilised various assessment tools in their diagnosis of FAIS. The ‘most commonly’ used tools, presented as mean ranks and proportions (in brackets) of those included in the survey, were special tests (3.4; 87%, 372/429), patient-reported signs/symptoms (3.4; 90%, 386/429), functional tests (3.4; 88%, 377/429), movement/range of motion (ROM) assessments (3.2; 77%, 332/429) and strength (2.9; 70%, 302/429). Information from balance assessments was the least utilised (2.0), followed by imaging (2.6) (Fig. 1).

Few physiotherapists reported being extremely confident (7%, 29/429) in diagnosing FAIS, with the majority either very (34%, 145/429) or moderately (46%, 198/429) confident. Less reported being slightly confident (11%, 48/429) and not at all confident (2%, 9/429).

#### Special tests

Special tests were the ‘most commonly’ used assessment tool with 87% (372/429) using these ‘always or often’ when diagnosing FAIS. Of those that utilised special tests, the flexion, adduction, internal rotation (FADIR) test was ‘always’ or ‘often’ used by 93% (393/424). Over 70% of respondents commonly used the flexion, abduction, external rotation (FABER) test (73%, 311/424) and the flexion, internal rotation test (74%, 314/424). Labral stress tests, or the Fitzgerald test were least commonly used (30%, 126/424) (Online Supplementary Material: Table and Fig. S1).

#### Patient-reported signs and symptoms

Motion-related hip pain was considered the most important clinical symptom in the diagnosis of FAIS and was used ‘always or often’ by 87% (373/428) of respondents, followed by position-related (83%, 354/428), groin (82%, 353/428) and anterior hip/thigh (77%, 329/428) pain. Buttock (31%, 134/428) and lateral hip (51%, 216/428) pain were considered the least important (Online Supplementary Material: Table and Fig. S2).

#### Functional tests

Functional tests were utilised ‘always’ or ‘often’ by 87% (372/429) of respondents. Of these, a bilateral squat (81%, 344/424) was most common followed by a single leg squat (79%, 335/424), gait analysis (71%, 302/424), single leg stance (67%, 282/424), and a hop or jump (54%, 229/424) (Online Supplementary Material: Table and Fig. S3).

#### Movement impairments/range of motion (ROM)

77% (332/429) of respondents utilised assessments of movement/ROM ‘always or often’ when diagnosing FAIS. The hip movement impairments deemed most important were internal rotation (94%, 399/424) and flexion (89%, 376/424) (Online Supplementary Material: Table and Fig. 4a). For assessment of movement/ROM impairments, visual estimations of joint ROM were most commonly reported (87%, 368/424), followed by muscle length tests (45%, 190/424), goniometry (26%, 111/424) and measurement tape (8%, 34/424) (Online Supplementary Material: Table and Fig. S4b).

#### Strength impairments

Most respondents regarded strength impairments as ‘always’ or ‘often’ important in their diagnosis (70%, 302/429). Of those that assessed strength, hip abduction was most important (76%, 320/421), followed by hip external rotation (65%, 273/421), hip extension (63%, 265/421), trunk/core strength (62%, 259/421), and hip flexion (61%, 257/421), adduction (51%, 215/421) and internal rotation (48%, 204/421) (Online Supplementary Material: Table and Fig. 5a). The ‘most commonly’ reported methods of strength assessment were manual muscle tests (77%, 324/421) and functional strength assessment (e.g., repetition maximum) (54%, 227/421). Isometric (e.g., handheld dynamometer, 24%; 101/421), isokinetic (6%, 25/421) and groin bar/force frame testing systems (5%, 22/421) were rarely used (Online Supplementary Material: Table and Fig. S5b).

#### Diagnostic imaging

Just over half (56%, 240/429) of respondents used imaging ‘always’ or ‘often’ in their clinical decision making. Radiographs (58%; 244/421), and magnetic resonance imaging (39%, 165/421) were most common. Computerised tomography (10%; 42/421), magnetic resonance arthrogram (23%, 97/421) and ultrasound (7%, 31/421) were used rarely (Online Supplementary Material: Table and Fig. S6).

#### Balance impairments

9% (37/429) of respondents reported that they never consider balance in their clinical decision making when determining a diagnosis of FAIS. One third (33%; 142/429) utilised balance assessments ‘always’ or ‘often’. The single-leg balance was considered the most important (60%, 236/392) and the star-excursion balance test (SEBT) the least (29%, 115/392) (Online Supplementary Material: Table and Fig. S7).

### Management of FAIS

**Fig. 2:**
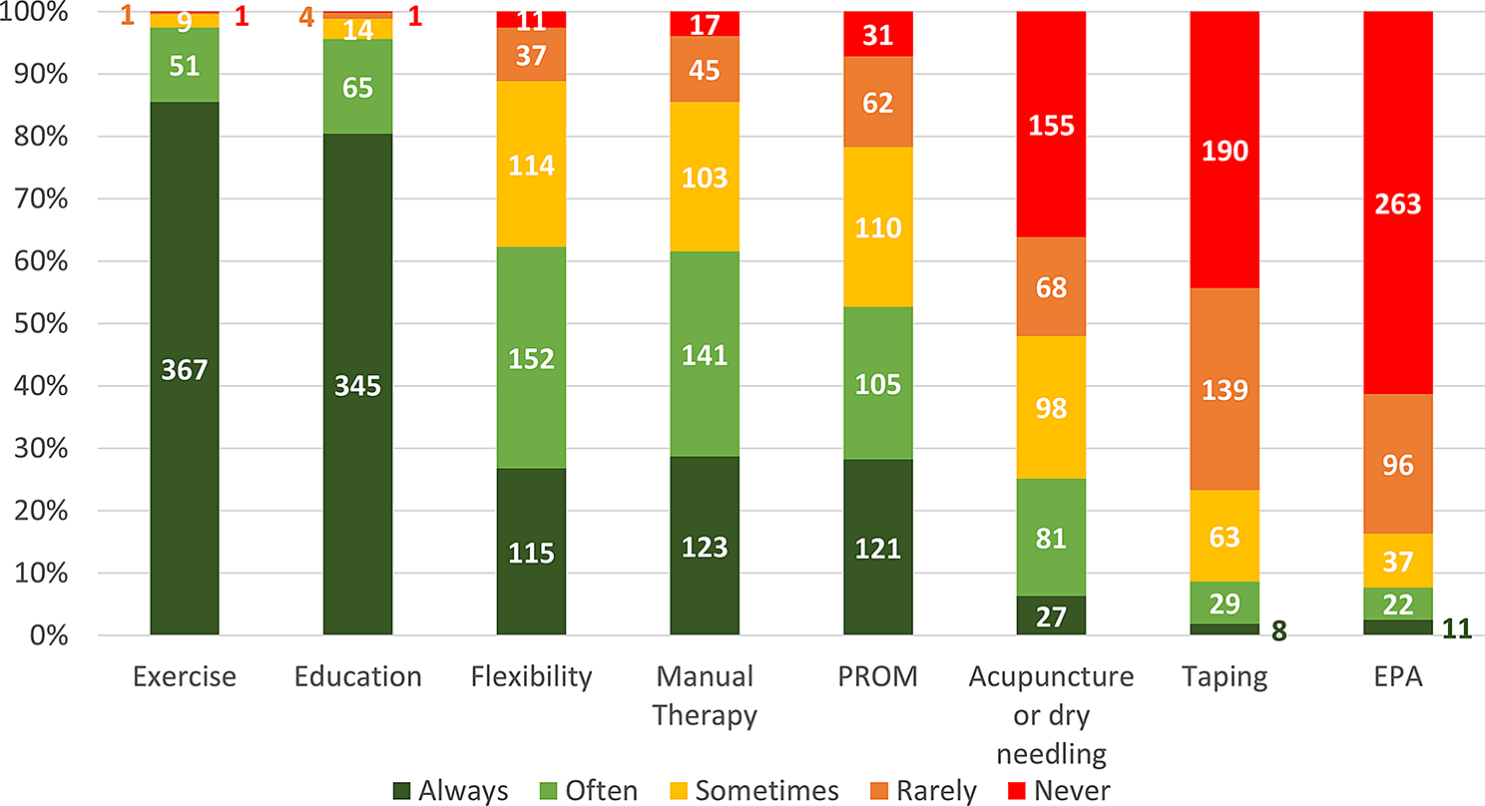
The frequency of interventions utilised in the management of FAIS

Nearly 80% (79%, 339/429) of physiotherapists reported treating patients across 4–10 treatment sessions, with few utilising less than four (9%, 40/429) or more than 16 (1%, 5/429) sessions. Most respondents were either very (34%; 145/429) or moderately (48%, 205/429) confident in their management of FAIS; a small number were extremely confident (8%, 36/429), or slightly (9%, 36/429) or not at all (0.9% 4/429) confident. Various modalities were employed in the management of FAIS, with the “most common” being exercise therapy (mean rank = 3.8; 97%; 418/429), patient education (3.8; 96%, 410/429), flexibility (e.g., ROM and stretching) (2.8; 62%, 267/429), manual therapy (2.7; 62%, 264/429), patient-reported outcome measures (PROM) (2.5; 52%, 226/429), and acupuncture/dry needling (1.4; 25%, 108/429). Taping (0.9; 9%, 37/429) and electrophysical agents (EPA) (0.7; 7%, 33/429) were rarely utilised (Fig. 2).

#### Exercise therapy

95% (407/429) of respondents use strengthening ‘always’ or ‘often’ as part of their exercise prescription. Neuromuscular control retraining (82%, 354/428), balance retraining (65%, 280/428) and cardiovascular exercise (43%, 183/428) were less commonly prescribed. When using strength training, 94% (401/426) targeted the hip abductors, and more than 70% strengthened the hip extensors (89%, 381/426), deep external rotators (73%, 311/426) and trunk muscles (75%, 320/426). Less emphasis was placed on the thigh (e.g., quadriceps and hamstrings), hip flexors (58% (248/426), and hip adductors (48%, 203/426) (Online Supplementary Material: Table and Fig. S8). Weight-bearing resistance exercises were used by most respondents (89%, 380/426) and most incorporated additional resistance (88%, 377/426). Non-weight bearing (54%, 228/426) or isometric (50%, 214/426) exercise, and plyometrics (46%, 195/426) were the least commonly reported strengthening option (Online Supplementary Material: Table and Fig. S9).

Functional movement retraining was most frequently used to address neuromuscular control in the management of FAIS (85%, 356/421). Proprioceptive retraining was utilised by two-thirds (67%, 281/421) of respondents, with walking and running gait retraining (59%, 248/421) and ballistic movement retraining (e.g., take-off and landing control) (52%, 221/421) least common (Online Supplementary Material: Table and Fig. S10).

#### Patient education

Nearly all the physiotherapists surveyed provided patient-specific education (96%, 410/429), most commonly advice on activity modification and load management (95%, 407/428). Many included advice on lifestyle modification (87%, 374/428) and pathomechanics of FAIS (83%, 354/428). Pain neuroscience was included in patient education by 54% (229/428) of respondents (Online Supplementary Material: Table and Fig. S11).

#### Flexibility

Flexibility, including joint ROM and stretching, was commonly included in management of FAIS by 62% (267/429) of respondents (Online Supplementary Material: Table and Fig. S12).

#### Manual therapy

62% of respondents (264/429) reported that they ‘always’ or ‘often’ use manual therapy as part of their management of FAIS. Of those that used manual therapy, massage (54%, 225/417) and mobilisation with movement (53%, 223/417) were the most common. Less than half used joint mobilisations (44%, 183/417) and muscle energy techniques (28%, 117/417) (Online Supplementary Material: Table and Fig. S13a, b, c).

#### Other interventions

Acupuncture/dry needling was rarely implemented with 25% (108/429) of physiotherapists reporting using this modality ‘always’ or ‘often’. Taping and EPA were not common, with only 9% (37/429) and 8% (33/429) of respondents, respectively, reporting they used these interventions commonly (Online Supplementary Material: Table and Fig. S12).

#### Patient-reported outcome measures (PROMs)

Approximately half (52%, 226/429) of respondents commonly utilised a PROM as part of their management of FAIS, and nearly a quarter (22%, 93/429) responded ‘rarely’ or ‘never’. Measures of patient-reported pain, including the visual analogue scale and numerical rating pain scale (74%, 292/397), and general function (i.e., Patient-Specific Functional Scale; 48%, 189/397) were the most commonly used PROM. The three most common hip/groin-specific measures were the Copenhagen Hip and Groin Outcome Score (HAGOS) (33%, 131/397), the Hip disability and Osteoarthritis Outcome Score (HOOS) (18%, 70/397) and the International Hip Outcome Tool (iHOT-33) (15%, 60/397) (Online Supplementary Material: Table and Fig. S14).

#### Referral

Physiotherapists would refer for non-physiotherapy management if; the patient was not responding to treatment (85%, 356/419), the patient requests onward referral (52%, 217/419), or if supported by assessment (30%, 126/419) or imaging findings (27%, 114/419) (Online Supplementary Material: Table and Fig. S15). Most physiotherapists (63%, 72/114) who were guided by imaging findings, indicated this was influenced by the presence of labral tears or altered hip-joint morphology (cam or pincer).

#### Non-physiotherapy interventions

**Fig. 3:**
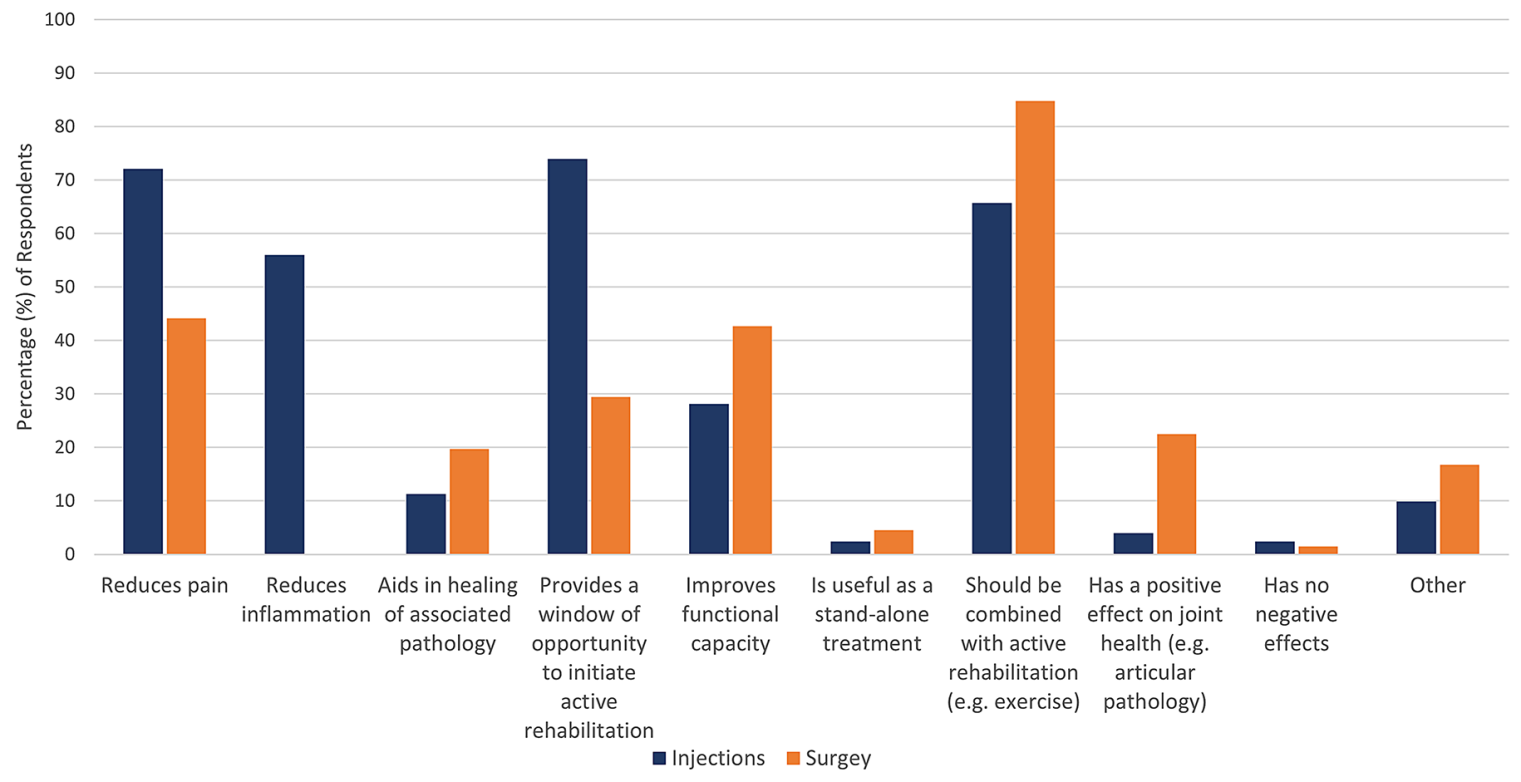
Physiotherapists’ beliefs regarding intra-articular injections and hip surgery

26% (111/428) of respondents refer patients with FAIS for non-physiotherapy interventions ‘always’ or ‘often’. Intra-articular injections and surgical interventions were commonly considered by 14% (60/418) and 16% (66/417) of physiotherapists respectively. The respondents who referred patients for intra-articular injections indicated that they believed this intervention will reduce pain (72%, 269/373) and inflammation (56%, 209/373), provide a window of opportunity to initiate active rehabilitation (74%, 276/373), and should always be provided in conjunction with an active rehabilitation programme (66%, 245/373). When referring a patient with FAIS for an intra-articular injection, respondents reported that corticosteroid injections were the most commonly used (77%, 286/373), followed by hyaluronic acid (18%, 69/373) and platelet rich plasma (15%, 56/373) injections. 15% of physiotherapists (56/373) were ‘not sure’ what type of intra-articular injection was used. Less than half felt surgery alone improved functional capacity (43%, 171/401), and 85% (340/401) felt that surgical interventions should be combined with active rehabilitation (Fig. 3).

#### Return to sport/work

Physiotherapists utilised many clinical tools when determining readiness for return to sport, work or other meaningful activity. Functional tests were the most common (93%, 397/426), followed by PROMs (77%, 329/426), strength (72%, 305/426) and ROM. Half of respondents (50%, 211/426) reported utilising a battery of tests to assess capability to return to pre-injury levels of activity (Online Supplementary Material: Table and Fig. S16).

## Discussion

To our knowledge, this study is the first to survey physiotherapists on their contemporary practice in FAIS diagnosis and management (see Supplementary Material 2). Although respondents reported a high level of confidence in their diagnosis and management of patients with FAIS, and current practice patterns are broadly consistent with current evidence-based guidelines, the use of assessment tools and intervention techniques that are inconsistent with contemporary evidence-based practice persists.

### Diagnosis of FAIS

The diagnosis of FAIS has recently been refined considerably, with current recommendations and expert opinion supporting a diagnostic triad of movement-related symptoms, clinical signs and imaging findings [1]. Consistent with these recommendations, movement-related symptoms were the most common diagnostic consideration of study respondents. Clinical signs of FAIS were included across several categories in our survey. Those considered most important were the FADIR special test and hip-joint ROM, particularly restrictions with internal rotation and hip flexion. Evidence of ROM impairments in people with FAIS is conflicting [12, 22], and currently, there is no agreement on the optimal methods of measuring ROM in this population [12]. Reduced hip internal rotation ROM and restricted movement with the bent knee fall-out test have been associated with the presence of cam morphology, seen in FAIS, but this has not been demonstrated in symptomatic populations [23]. The FADIR test has high levels of sensitivity and specificity in diagnosing intra-articular pathology more broadly [17], but is best utilised to rule out intra-articular pathology, including FAIS [17, 24]. This study demonstrated that a high proportion of physiotherapists utilise other special tests (e.g., FABER, and the flexion, internal rotation test) in their diagnosis of FAIS, but evidence is lacking to support their use for this purpose [25].

Despite imaging findings being the third component of the diagnostic triad for FAIS [1], imaging uptake was relatively low, with x-ray utilised commonly by approximately half of participants and MRI by one-third. Associated barriers, reported by respondents included cost, limited or no access, or legislative requirements limiting physiotherapist’s ability to direct refer for imaging within specific countries. A small number of respondents also considered that imaging alone does not provide any additional diagnostic utility, which may also partially explain its limited use. Expert opinion suggests that when used in isolation, imaging has limited diagnostic value in FAIS and cannot be used to determine the primary cause of symptoms [17]. In addition, given the frequency of radiological signs of FAIS in asymptomatic populations [26, 27], it is recommended that imaging findings should only be used in conjunction with relevant clinical signs and symptoms [17].

Strength testing was utilised by 70% of physiotherapists in their assessment of FAIS despite 95% including strengthening interventions in their management. Previous investigations of strength deficits in FAIS have demonstrated impairments in hip adduction, abduction, flexion, extension, internal rotation and external rotation [12, 28], which aligns with the assessment priorities stated by physiotherapists in this study. With strength impairments often being small in this population [29], the importance of accurate, objective assessments of strength to guide targeted interventions is vital [12]. Interestingly, the use of reliable, objective measures to assess hip and lower limb strength was not routinely used by respondents, with only a quarter regularly using handheld dynamometry, which has demonstrated reliability in this context using both manual and external belt fixation [30–32]. In contrast, manual muscle tests are used by over three-quarters of respondents despite questionable suitability for measuring muscle strength in populations with small impairments [33, 34].

### Management

Most physiotherapists reported a moderate to high level of confidence in their management of FAIS. Despite this, there were noticeable variations between current practice and best evidence recommendations. Consistent with current expert recommendations for the management of FAIS and, more broadly, hip and groin pain [1, 4], physiotherapy management priorities were exercise, including functional movement retraining, and education. Symptom management using manual modalities and adjunct therapies (e.g., acupuncture, taping and electrotherapeutic modalities) was less common. Exercise therapy has repeatedly been promoted as the central component of management in this population [4, 35], with a focus on weight-bearing strengthening exercises targeting hip abduction, extension, and external rotation. It has been recommended that physiotherapy treatment of FAIS be of sufficient duration (e.g., 3 months) to allow physiological adaptations, including strength [35]. The majority of survey respondents indicated they routinely provided 4–10 treatment sessions. These sessions could conceivably be spread over 3 months, but lower numbers suggest that at least a portion of these patients would not be receiving a sufficient treatment duration or guidance to realise some of the targeted physiological adaptations. Incorporating treatment and exercise parameters which provide adequate opportunity for clinical and physiological adaptations to occur should be considered when physiotherapists are prescribing exercise for patients with FAIS. While we did not ask about the dosage of strength exercises, this is an important discussion for future studies, particularly to explore the impact of dosage on exercise effect [36].

Despite evidence that individuals with FAIS exhibit deficits in balance [37] and dynamic movements [38], and that cardiovascular fitness should be maintained in this population [4, 11], there was noticeably less emphasis on these treatment priorities by physiotherapists. All of these factors have been recommended as important components of return-to-sport [11] for patients with FAIS and should be incorporated into treatment planning. Further, recent evidence suggests that people with FAIS seek out treatment from physiotherapists to address goals related to exercise, including a desire for less pain and greater confidence when performing their preferred activity. Physiotherapists should ensure they are meeting the needs and expectations of their patients by including guidance on how to exercise and re-engage in physical activity without pain [10].

Patient education, with a focus on activity modification and load monitoring, was considered a core component of physiotherapy management in this survey (95% of survey respondents), aligning with current best-evidence recommendations [11] and patients’ expectations [39]. Education relating to the pathomechanics of FAIS was commonly provided, but education addressing pain neurophysiology was less routine. The pathomechanics of FAIS centring on abutment of the acetabular rim on the femoral head neck junction has been a popular explanation for radiological changes seen in this population [40]. However, the relationship between joint mechanics, radiological changes and symptoms has recently been brought into question with evidence demonstrating that a large proportion of asymptomatic athletes display similar radiological findings to activity-matched peers, suggesting other potential drivers of symptoms [27]. Pain neurophysiology in FAIS has not been explored in detail, but the lack of clarity around the source of symptoms in this population supports the need for further investigation.

Although respondents reported a high level of confidence in their management, PROMs to assess the effectiveness of treatment were used by just over half of physiotherapists (52%, 226/429). Most respondents who used PROMs only measured symptoms (i.e., numerical pain rating scale) or global function (i.e., Patient Specific Functional Scale), with efforts to quantify the perceived impact of FAIS on patients’ health and quality of life, including how this changed with treatment, rarely utilised. The HAGOS and iHOT-33, despite being used by less than a fifth of physiotherapists, are valid and reliable measures in young active people with hip and groin pain, including FAIS [41, 42] and should be incorporated into a comprehensive assessment and management plan.

Manual therapy was the most commonly used adjunctive therapy, aligning with expert recommendations to manage symptoms [4]. There is currently no evidence to support the use of acupuncture, dry needling, or electrotherapy in the management of FAIS, which was reflected in this study by the low levels of reported use. A large proportion of physiotherapists (85%) reported referring for non-physiotherapy management when a patient is not responding to treatment, with a quarter of respondents commonly referring for surgical or intra-articular injections. To date, no trials have compared surgery and physiotherapy-led conservative care as equal interventions. In the randomised controlled trials completed, the comparator has always been ‘more physiotherapy’, or ‘more tailored physiotherapy’, in people who have already ‘failed’ with an initial course of physiotherapy-led conservative care [43, 44]. Consequently, there is debate over whether surgery or conservative care provides superior outcomes for patients with FAIS in both the short and long term [36]. With respect to the use of intra-articular injections in the management of FAIS, research is limited and often conflicting [45, 46], which may somewhat account for the low proportion of physiotherapists in this survey considering this an option.

There are some limitations to this study. While every attempt was made to ensure a high response rate (e.g., by resending emails/reminders, extending the survey time, and including an incentive), the response was less than half of what we anticipated, introducing bias [47]. The low response rate may be partially attributable to the primary method of recruitment. Participants were initially recruited by email through national physiotherapy organisations in each of the target countries. This approach resulted in a slower than expected rate of recruitment. Social media advertising was utilised to increase the response rate from the chosen sample population, but it is not clear what effect this had on the overall number of responses received. The high proportion of respondents reporting that they were members of their national association (95%) suggests that recruitment directly to physiotherapy organisations contributed the largest number of participants. Primary recruitment through national physiotherapy organisations may have biased our sample, as it likely included physiotherapists who were engaged in their profession and, therefore, were more likely to be aware of consensus recommendations and other contemporary best evidence. Most respondents worked in private practice, which could influence specific results such as duration of care and access to imaging modalities. Additionally, the low number of respondents from specific countries (e.g., Canada), makes it impossible to determine if the responses from these populations were reflective of the wider physiotherapy population. It also limits the ability to ascertain specific differences between countries in the diagnosis and management of FAIS.

## Conclusion

This is the first multi-national survey of practising physiotherapists to examine contemporary diagnosis and management of FAIS. Based on a convenience sample from six countries, physiotherapists broadly utilise the recommended diagnostic triad of symptoms, clinical signs, and imaging findings to guide their clinical decision-making, with some variation around what symptoms and clinical signs are incorporated. Management is predominantly focused on exercise interventions, in agreement with current consensus recommendations, although assessments that guide these interventions, particularly strength and ROM, are not usually undertaken using reliable measures. Monitoring the efficacy of physiotherapy interventions is rarely included in contemporary practice. Patient education is provided by nearly all physiotherapists, but the content is inconsistent. Given the ability for physiotherapists to influence patients’ health beliefs and outcomes, further research on patient education is required to better understand the optimal components to emphasise.

## Electronic supplementary material

Below is the link to the electronic supplementary material.

Supplementary Material 1

Supplementary Material 2

## Data Availability

Data is provided within the manuscript or supplementary information files.

## Abbreviations

EPA: Electrophysical Agents
FABER: Flexion Abduction External Rotation
FADIR: Flexion Adduction Internal Rotation
FAIS: Femoroacetabular Impingement Syndrome
HAGOS: Hip and Groin Outcome Score
HOOS: Hip Disability and Osteoarthritis Outcome Score
iHOT-33: International Hip Outcome Tool
OA: Osteoarthritis
PROM: Patient-reported Outcome Measure
ROM: Range of Motion
SEBT: Star Excursion Balance Test
STROBE: Strengthening the Reporting of Observational Studies in Epidemiology
