# Supplementary material for: Diagnosis and management of femoroacetabular impingement syndrome (FAIS): a survey of contemporary physiotherapy practice
Source: BMC Musculoskelet Disord. 2025 Oct 7;26:924. doi: 10.1186/s12891-025-08708-7 (PMC12505584; doi:10.1186/s12891-025-08708-7)

**SUPPLEMENTARY MATERIAL 1 – DIAGNOSIS AND MANAGEMENT OF FAIS**

**DIAGNOSIS**

**Table S1. Special tests**

| Frequency of use | FADIR test | FABER test | Hip flex-int rot test | Hip quadrant (Scour test) | Int rotation-flexion-axial compression test | Thomas test | Fitzgerald test/labral stress test |
| --- | --- | --- | --- | --- | --- | --- | --- |
| Always | 272 | 165 | 161 | 114 | 77 | 72 | 47 |
| Often | 121 | 146 | 153 | 136 | 121 | 98 | 79 |
| Sometimes | 24 | 86 | 84 | 98 | 109 | 144 | 124 |
| Rarely | 7 | 24 | 20 | 51 | 74 | 82 | 87 |
| Never | 0 | 3 | 6 | 25 | 43 | 28 | 87 |

**Figure S1. Special tests**


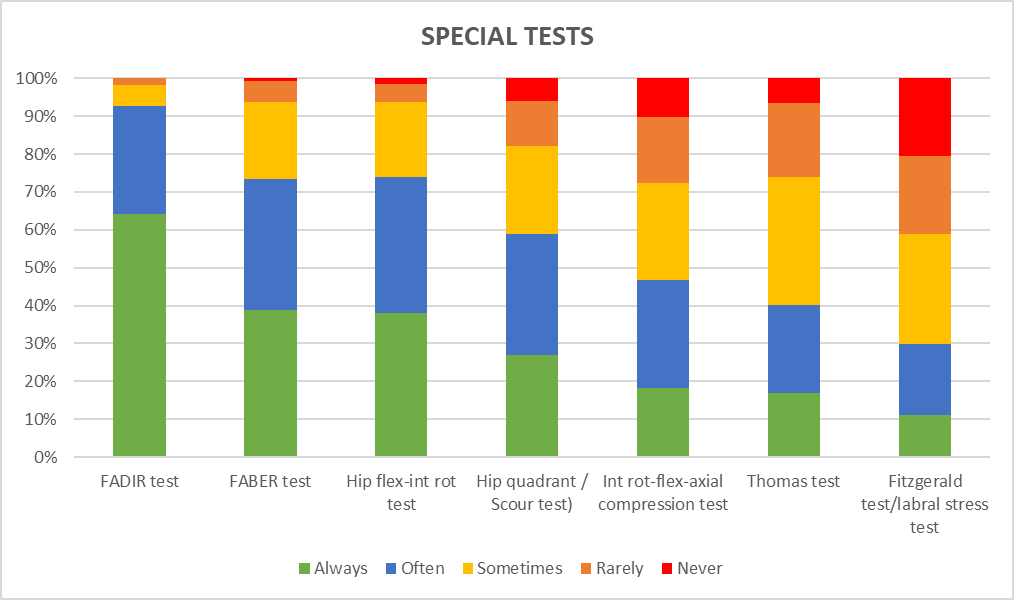


**Table S2. Patient-reported signs and symptoms**

| Frequency of use | Motion-related hip pain | Position-related hip pain | Stiffness | Groin pain | Mechanical symptoms | Anterior hip/thigh pain | Lateral hip pain | Buttock pain |
| --- | --- | --- | --- | --- | --- | --- | --- | --- |
| Always | 183 | 161 | 153 | 146 | 130 | 124 | 78 | 40 |
| Often | 190 | 193 | 197 | 207 | 169 | 205 | 138 | 94 |
| Sometimes | 47 | 58 | 65 | 69 | 102 | 91 | 134 | 194 |
| Rarely | 7 | 16 | 12 | 5 | 25 | 6 | 66 | 91 |
| Never | 1 | 0 | 1 | 1 | 2 | 2 | 12 | 9 |

**Figure S2. Patient-reported signs and symptoms**


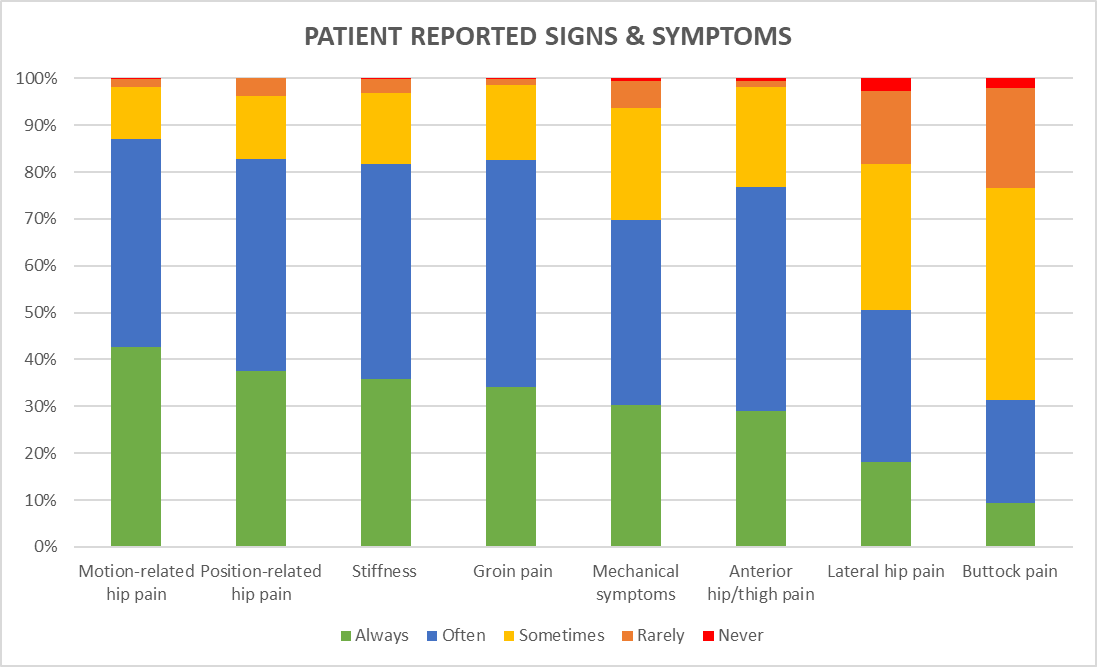


**Table S3. Functional tests**

| Frequency of use | Bilateral squat | Single-leg squat | Gait analysis (walking/running) | Single-leg stance | Hop or jump |
| --- | --- | --- | --- | --- | --- |
| Always | 194 | 167 | 165 | 154 | 82 |
| Often | 150 | 168 | 137 | 128 | 147 |
| Sometimes | 58 | 74 | 92 | 92 | 136 |
| Rarely | 19 | 11 | 23 | 41 | 50 |
| Never | 3 | 4 | 7 | 9 | 9 |

**Figure S3. Functional tests**


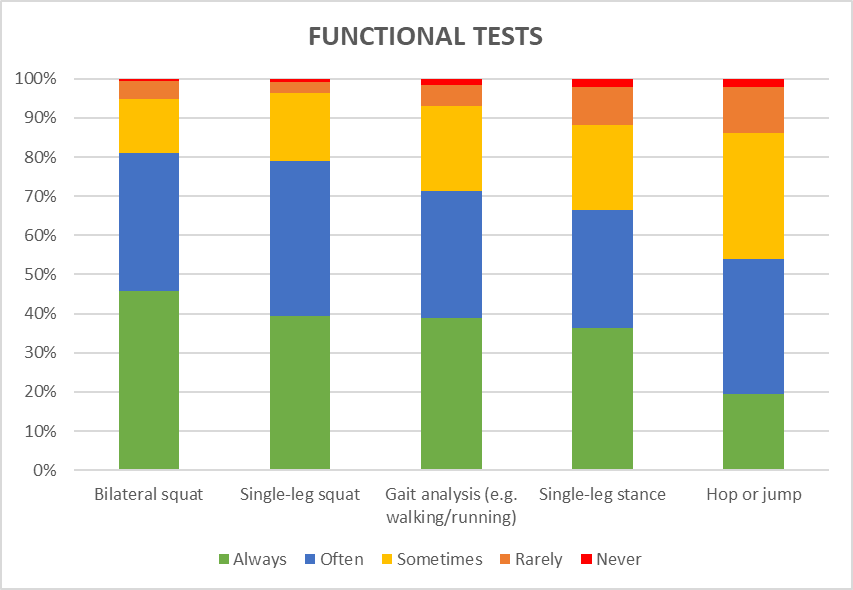


**Table S4a. Movement and range of motion**

| Frequency of use | Hip internal rotation | Hip flexion | Hip external rotation | Hip adduction | Hip abduction | Hip extension |
| --- | --- | --- | --- | --- | --- | --- |
| Always | 244 | 206 | 103 | 87 | 53 | 48 |
| Often | 155 | 170 | 129 | 124 | 126 | 97 |
| Sometimes | 24 | 48 | 192 | 204 | 240 | 274 |
| Rarely | 0 | 0 | 0 | 0 | 0 | 0 |
| Never | 1 | 0 | 0 | 9 | 5 | 5 |

**Figure S4a. Movement and range of motion**


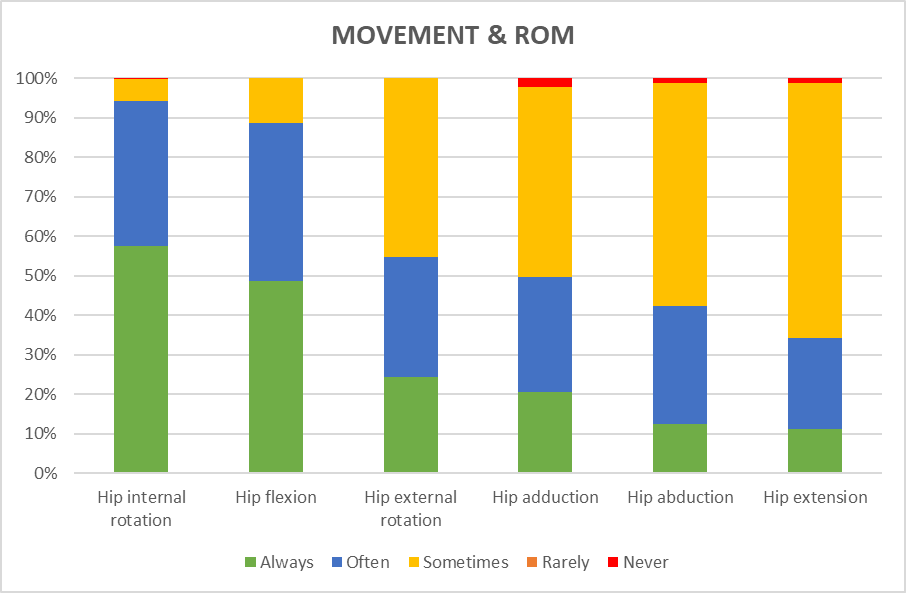


**Table S4b. Methods of assessing movement and range of motion impairments**

| Frequency of use | Visual estimation of joint range of motion | Muscle length tests | Goniometry | Tape measure |
| --- | --- | --- | --- | --- |
| Always | 198 | 73 | 32 | 6 |
| Often | 170 | 117 | 79 | 28 |
| Sometimes | 36 | 149 | 117 | 73 |
| Rarely | 17 | 62 | 127 | 170 |
| Never | 3 | 23 | 69 | 147 |

**Figure S4b. Methods of assessing movement and range of motion impairments**


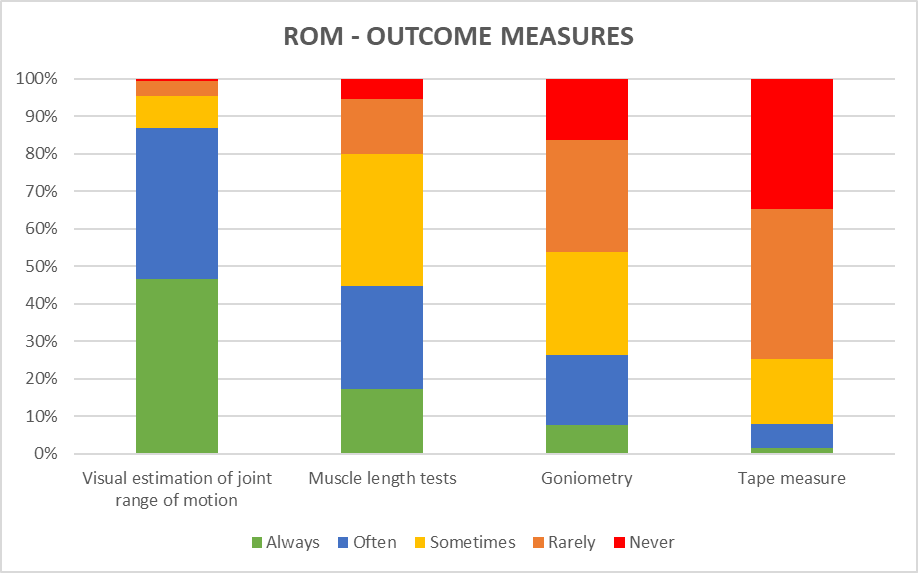


**Table S5a. Strength impairments**

| Frequency of use | Hip abduction | Trunk/core | Hip external rotation | Hip extension | Hip flexion | Hip adduction | Hip internal rotation |
| --- | --- | --- | --- | --- | --- | --- | --- |
| Always | 165 | 130 | 124 | 119 | 115 | 95 | 79 |
| Often | 155 | 129 | 149 | 146 | 142 | 120 | 125 |
| Sometimes | 74 | 110 | 113 | 104 | 129 | 117 | 152 |
| Rarely | 22 | 40 | 28 | 45 | 31 | 78 | 55 |
| Never | 5 | 12 | 7 | 7 | 4 | 11 | 10 |

**Figure S5a. Strength impairments**


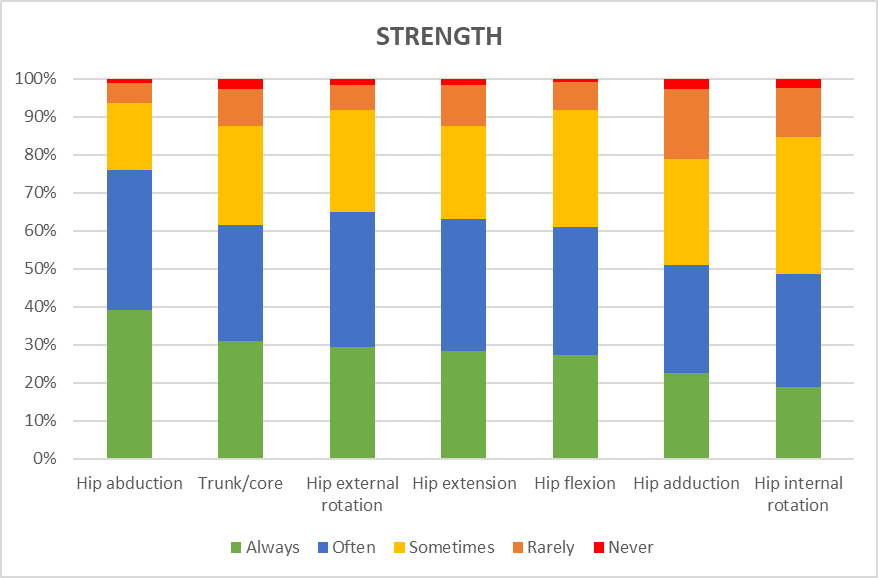


**Table S5b. Methods of strength assessment**

| Frequency of use | Manual muscle tests (e.g., Oxford Scale) | Dynamic/functional assessment (e.g., repetition maximum) | Handheld dynamometry | Isokinetic dynamometry | Groin bar/force frame testing system |
| --- | --- | --- | --- | --- | --- |
| Always | 199 | 72 | 40 | 9 | 9 |
| Often | 125 | 155 | 61 | 16 | 13 |
| Sometimes | 52 | 107 | 44 | 22 | 25 |
| Rarely | 27 | 34 | 80 | 70 | 58 |
| Never | 18 | 53 | 196 | 304 | 316 |

**Figure S5b. Methods of strength assessment**


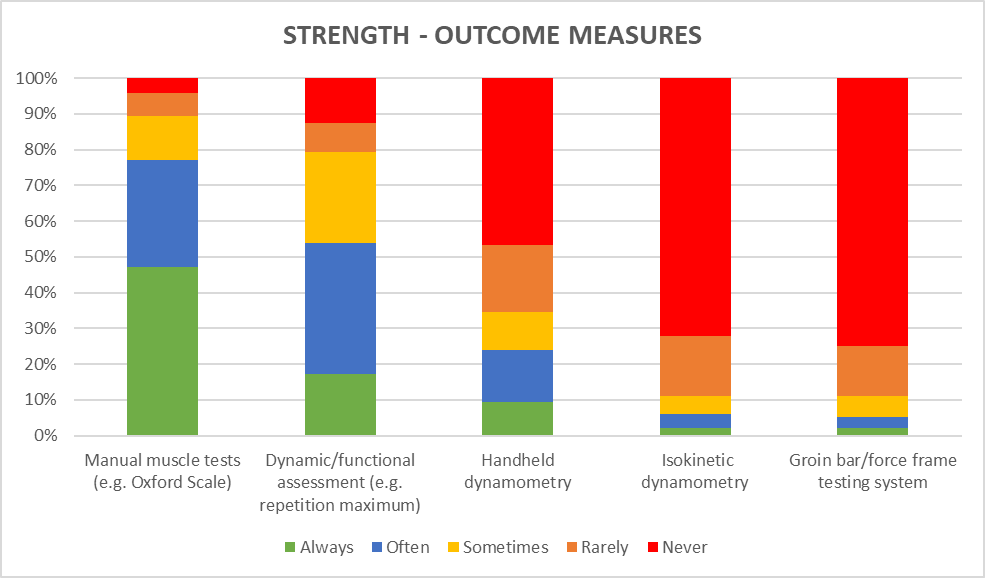


**Table S6. Diagnostic imaging**

| Frequency of use | X-ray | Magnetic resonance imaging | Magnetic resonance arthrogram | Computed tomography | Ultrasound |
| --- | --- | --- | --- | --- | --- |
| Always | 92 | 43 | 29 | 10 | 9 |
| Often | 152 | 122 | 68 | 32 | 22 |
| Sometimes | 124 | 151 | 79 | 88 | 42 |
| Rarely | 33 | 70 | 110 | 142 | 132 |
| Never | 20 | 35 | 135 | 149 | 216 |

**Figure S6. Diagnostic imaging**


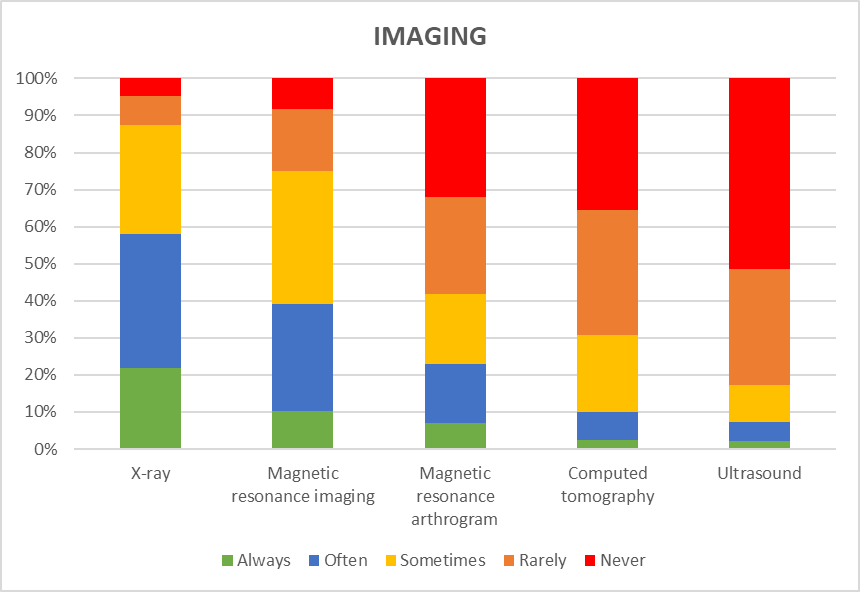


**Table S7. Balance impairments**

| Frequency of use | Single-leg balance | Star excursion balance test (SEBT) |
| --- | --- | --- |
| Always | 112 | 26 |
| Often | 124 | 89 |
| Sometimes | 98 | 126 |
| Rarely | 56 | 80 |
| Never | 2 | 71 |

**Figure S7. Balance impairments**

**
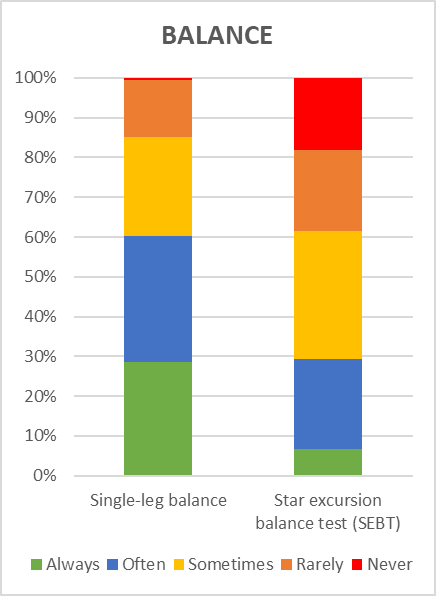
**

**MANAGEMENT**

**Table S8. Muscles/movements targeted**

| Frequency of use | Hip abduction | Hip extension | Trunk | Hip external rotation | Thigh | Hip flexion | Hip adduction |
| --- | --- | --- | --- | --- | --- | --- | --- |
| Always | 263 | 229 | 157 | 155 | 97 | 89 | 91 |
| Often | 138 | 152 | 163 | 156 | 151 | 159 | 112 |
| Sometimes | 21 | 39 | 81 | 89 | 139 | 127 | 119 |
| Rarely | 2 | 4 | 16 | 20 | 32 | 44 | 89 |
| Never | 2 | 2 | 9 | 6 | 7 | 7 | 15 |

**Figure S8. Muscles/movements targeted**

**
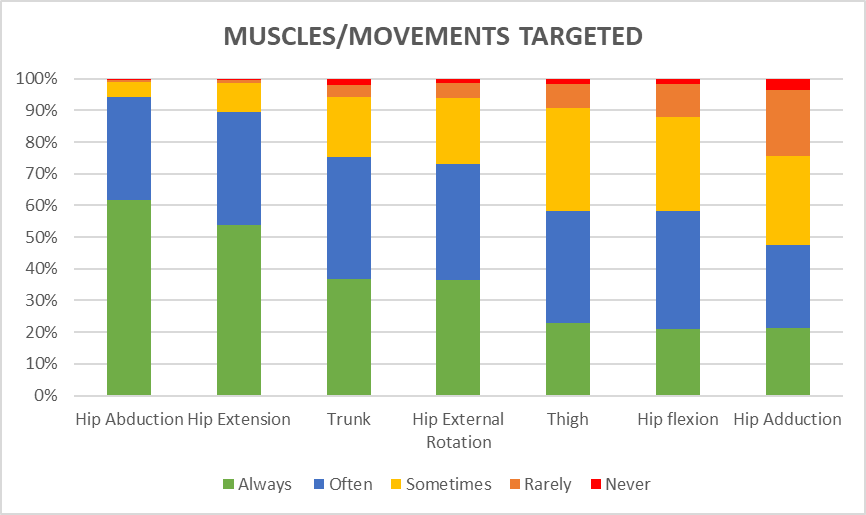
**

**Table S9. Strengthening methods**

| Frequency of use | Weight bearing | Resistance | Non-weight bearing | Isometric | Plyometrics |
| --- | --- | --- | --- | --- | --- |
| Always | 212 | 183 | 79 | 67 | 73 |
| Often | 168 | 194 | 149 | 147 | 122 |
| Sometimes | 40 | 39 | 152 | 144 | 137 |
| Rarely | 4 | 6 | 38 | 53 | 71 |
| Never | 2 | 4 | 8 | 15 | 23 |

**Figure S9. Strengthening methods**

**
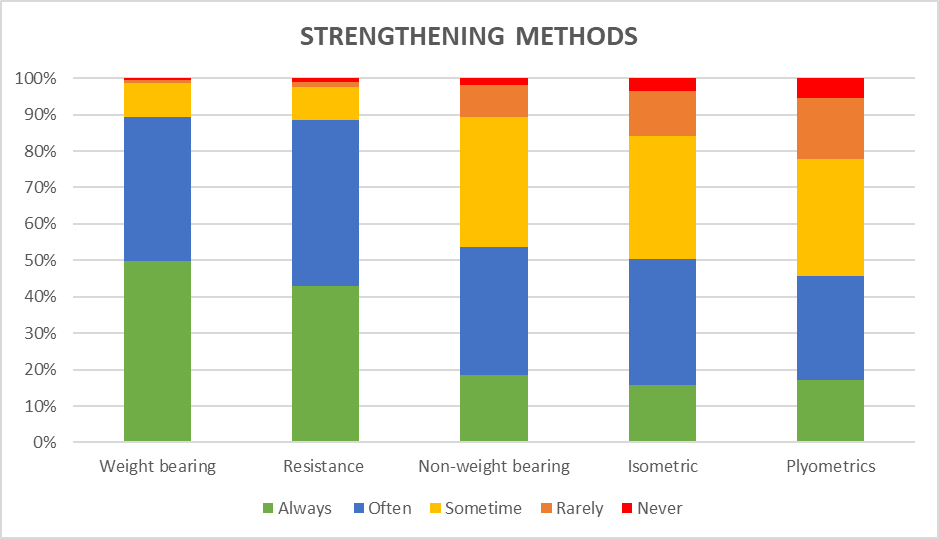
**

**Table S10. Methods for neuromuscular control**

| Frequency of use | Functional movement retraining (e.g., sitting, standing, squatting) | Proprioceptive retraining | Gait retraining (e.g., walking, running) | Ballistic movement retraining (e.g., landing control) |
| --- | --- | --- | --- | --- |
| Always | 204 | 116 | 103 | 77 |
| Often | 152 | 165 | 145 | 144 |
| Sometimes | 51 | 93 | 120 | 141 |
| Rarely | 12 | 42 | 42 | 51 |
| Never | 2 | 5 | 11 | 8 |

**Figure S10. Methods for neuromuscular control**

**
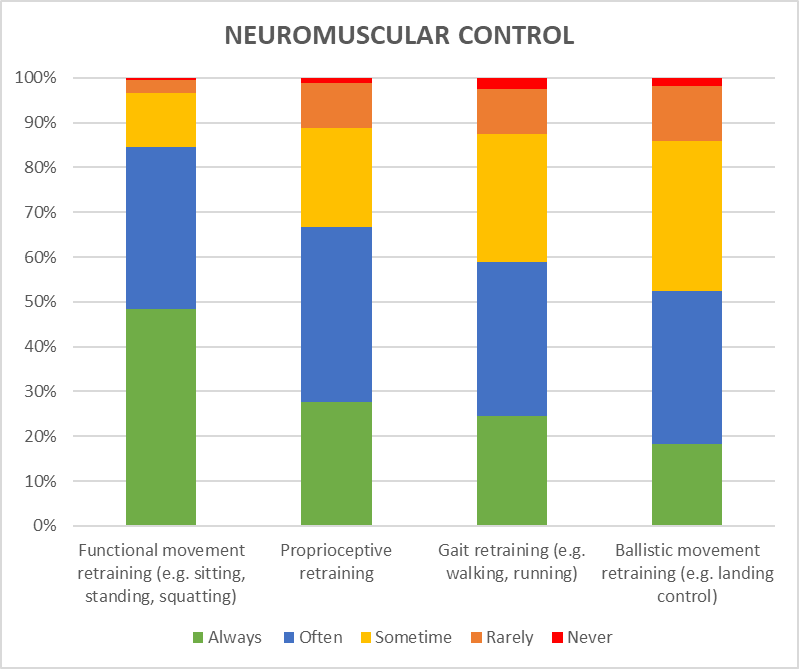
**

**Table S11. Patient education**

| Frequency of use | Activity modification/load management | Lifestyle modification | Pathoanatomical theory of FAIS | Pain neuroscience |
| --- | --- | --- | --- | --- |
| Always | 315 | 243 | 211 | 91 |
| Often | 92 | 131 | 143 | 138 |
| Sometimes | 18 | 51 | 56 | 148 |
| Rarely | 2 | 3 | 15 | 42 |
| Never | 1 | 0 | 3 | 9 |

**Figure S11. Patient education**


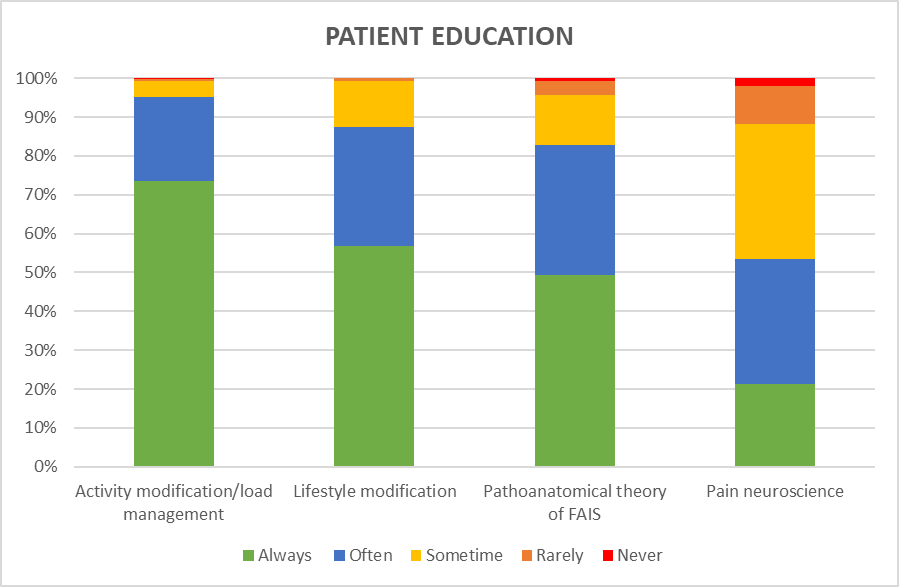


**Table S12. Other management**

| Frequency of use | Flexibility (e.g., joint range of motion and/or stretching) | Acupuncture or dry needling | Taping | Electrophysical agents (e.g., electrotherapy, thermal) |
| --- | --- | --- | --- | --- |
| Always | 115 | 27 | 8 | 11 |
| Often | 152 | 81 | 29 | 22 |
| Sometimes | 114 | 98 | 63 | 37 |
| Rarely | 37 | 68 | 139 | 96 |
| Never | 11 | 155 | 190 | 263 |

**Figure S12. Other management**

**
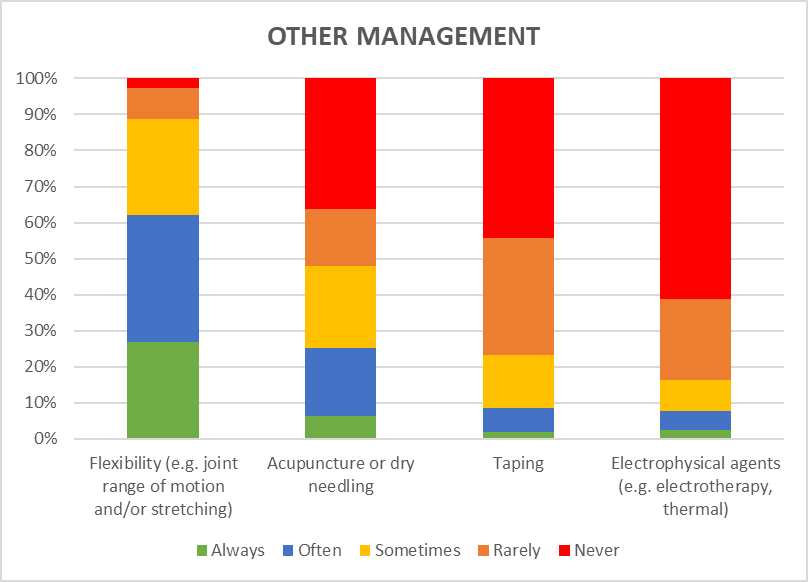
**

**Table S13a. Manual therapy**

| Frequency of use | Massage therapy (e.g., soft tissue massage, trigger point massage) | Mobilisation with movement | Joint mobilisation (e.g., Maitland, Kaltenborn) | Muscle energy techniques |
| --- | --- | --- | --- | --- |
| Always | 92 | 70 | 62 | 32 |
| Often | 133 | 153 | 121 | 85 |
| Sometimes | 112 | 108 | 94 | 107 |
| Rarely | 46 | 53 | 83 | 85 |
| Never | 29 | 28 | 52 | 103 |

**Figure S13a. Manual therapy**

**
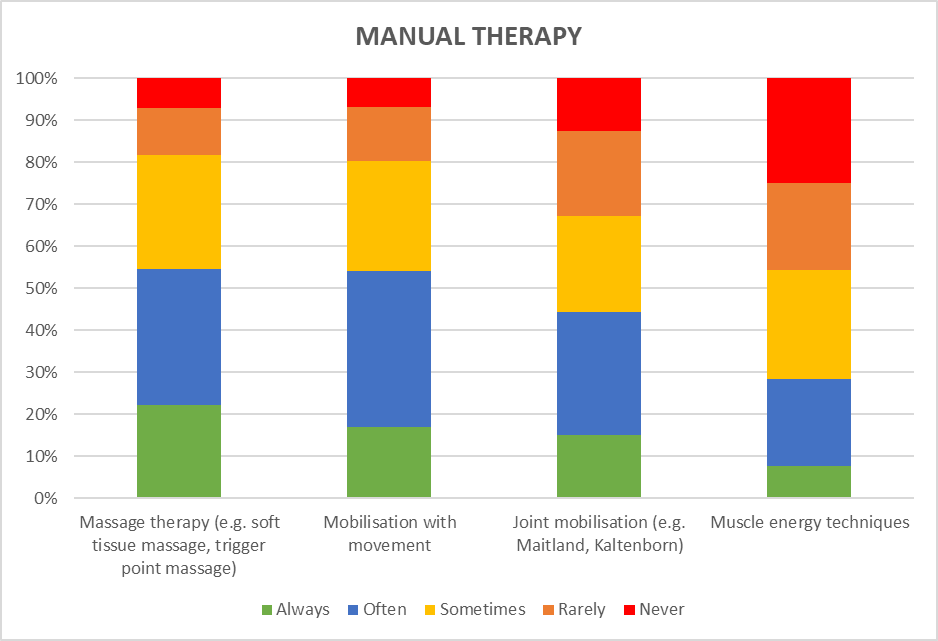
**

**Table S13b. Massage treatment area**

| Frequency of use | Gluteal region | Hip flexors | Hip adductors | Thigh | Lumbar region |
| --- | --- | --- | --- | --- | --- |
| Always | 100 | 68 | 55 | 41 | 41 |
| Often | 174 | 159 | 123 | 109 | 102 |
| Sometimes | 76 | 100 | 115 | 128 | 127 |
| Rarely | 27 | 41 | 78 | 83 | 83 |
| Never | 6 | 15 | 12 | 22 | 30 |

**Figure S13b. Massage treatment area**

**
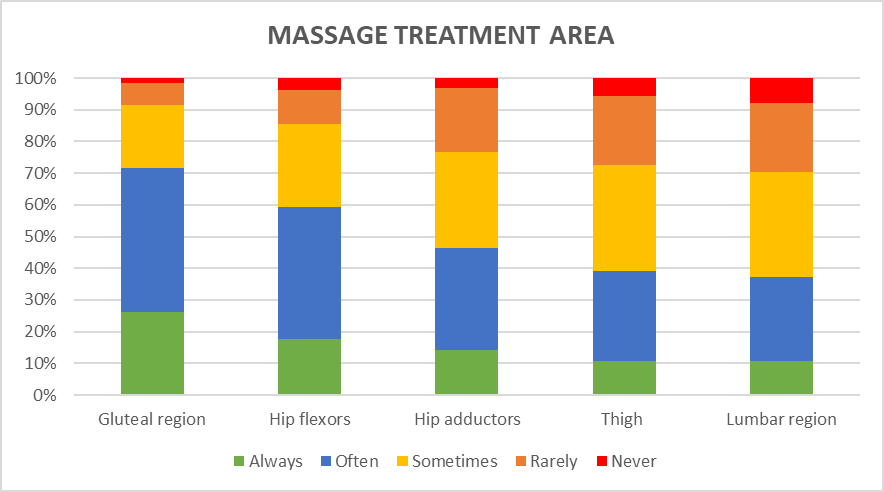
**

**Table S13c. Mobilisation treatment area**

| Frequency of use | Hip joint | Lumbar spine | Sacroiliac joint | Knee joint |
| --- | --- | --- | --- | --- |
| Always | 145 | 37 | 36 | 8 |
| Often | 148 | 103 | 65 | 24 |
| Sometimes | 75 | 144 | 100 | 87 |
| Rarely | 24 | 69 | 105 | 153 |
| Never | 3 | 42 | 89 | 123 |

**Figure S13c. Mobilisation treatment area**

**
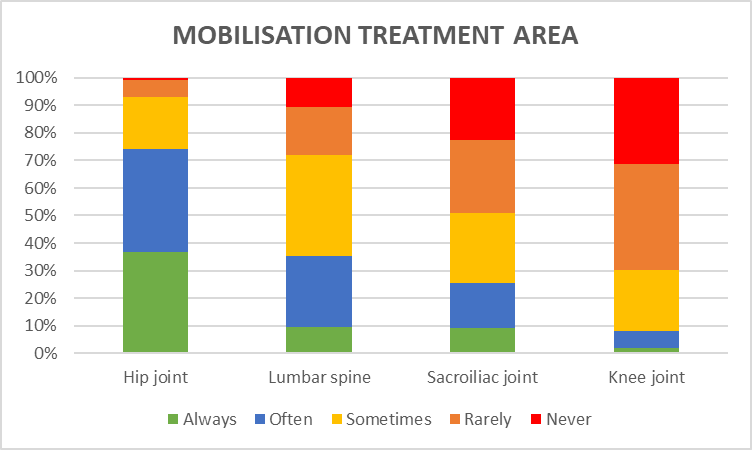
**

**Table S14. Patient-reported outcome measures**

| Patient-reported outcome measure | Total | Percentage |
| --- | --- | --- |
| Visual Analogue Scale/Numerical Rating Scale | 292 | 74 |
| Patient Specific Functional Scale (PSFS) | 189 | 48 |
| Copenhagen Hip and Groin Outcome Score (HAGOS) | 131 | 33 |
| Hip disability and Osteoarthritis Outcome Score (HOOS) | 70 | 18 |
| International Hip Outcome Tool – 33 Item (IHOT-33) | 60 | 15 |
| Global rating of change scale (GROC) | 48 | 12 |
| Westen Ontario and McMaster University Osteoarthritis Index (WOMAC) | 47 | 12 |
| Hip Outcome Score (HOS) | 41 | 10 |
| Modified Harris Hip Score (MHHS) | 26 | 7 |
| Other (i.e., LEFS, Obero, MSK-HQ and VISA G) | 25 | 6 |
| Non-arthritic hip score (NAHS) | 15 | 4 |

**Figure S14. Patient-reported outcome measures**

**
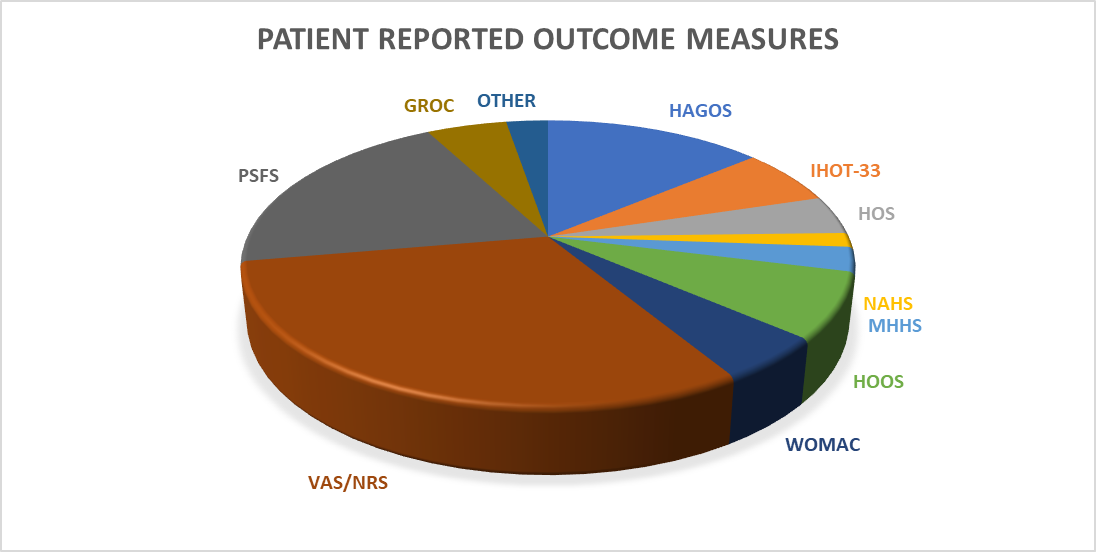
**

**Table S15. Referral**

| Criteria for onward referral | Total | Percentage |
| --- | --- | --- |
| Patient not responding to treatment ^a^ | 356 | 84.96420048 |
| Patients request | 217 | 51.78997613 |
| Specific assessment findings | 126 | 30.07159905 |
| Imaging findings | 114 | 27.20763723 |
| Other | 51 | 12.17183771 |
| Conclusion of funded treatments | 24 | 5.727923628 |
| Patient not responding to treatment | 356 | 84.96420048 |

**^a^** Timeframes for referral: Respondents specified that they would wait for an average of 6 ± 3 sessions before referring a patient if they were not responding. Forty-four respondents preferred to use time rather than treatment sessions to determine the threshold for onward referral. They reported an average time of 17 ± 7 weeks before referring a patient if they were not responding.

**Figure S15. Referral**

**
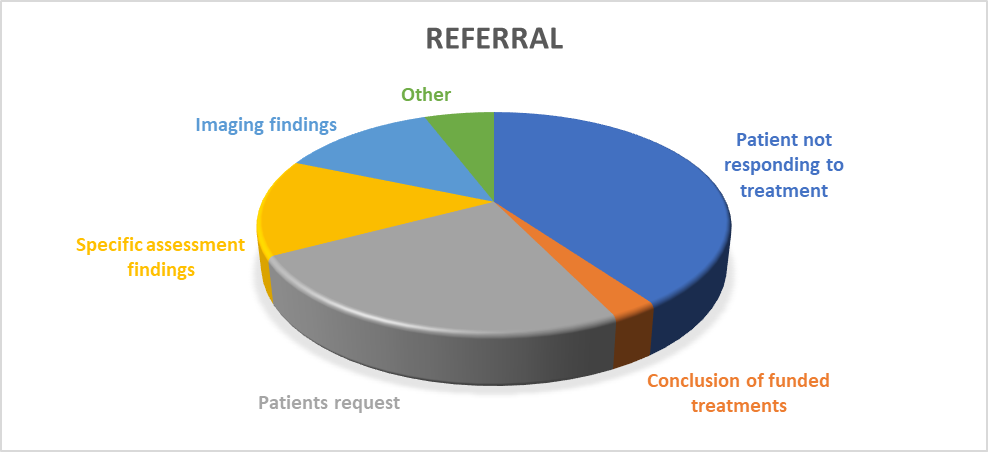
**

**Table S16: Return to sport/work**

| Clinical tools to determine readiness for return to sport/work | Total | Percentage |
| --- | --- | --- |
| Functional tests/screening (e.g., performance of patient’s meaningful task) | 397 | 93.19248826 |
| Patient-reported outcome measures | 329 | 77.23004695 |
| Lower limb strength equivalent to the contralateral limb | 305 | 71.59624413 |
| Pain-free range of motion | 241 | 56.57276995 |
| Completion of patient-specific movement battery | 211 | 49.53051643 |
| Other | 13 | 3.051643192 |

**Figure S16. Return to sport/work**


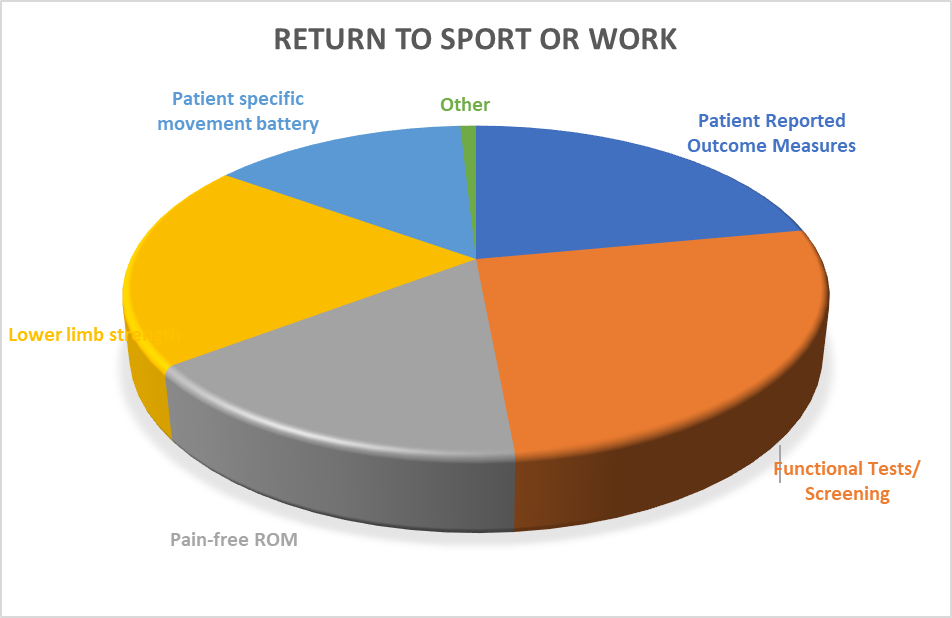

Supplement: Supplementary file 1 — Supplementary Material 1 [file 12891_2025_8708_MOESM1_ESM.docx]
