# Supplementary material for: Diagnosis and management of femoroacetabular impingement syndrome (FAIS): a survey of contemporary physiotherapy practice
Source: BMC Musculoskelet Disord. 2025 Oct 7;26:924. doi: 10.1186/s12891-025-08708-7 (PMC12505584; doi:10.1186/s12891-025-08708-7)
Supplement: Supplementary file 2 — Supplementary Material 2 [file 12891_2025_8708_MOESM2_ESM.docx]

**SUPPLEMENTARY MATERIAL 2 – SURVEY**

**Diagnosis and management of Femoroacetabular Impingement Syndrome (FAIS): An international survey of current physiotherapy practice**

Thank you for your interest in participating in this study. We are seeking physiotherapists who are actively involved in treating patients with hip-related pain in their clinical practice, to complete a survey on clinical diagnosis and contemporary physiotherapy management of femoroacetabular impingement syndrome (FAIS).

Femoroacetabular impingement syndrome is the most common cause of hip-related pain in young active adults, evident in nearly 50% of young adults presenting with hip pain. It is characterised by motion-related hip pain and altered bony morphology of the hip joint, thought to contribute to the development of pain, intra-articular pathology and the early onset of hip osteoarthritis. Physiotherapist-led conservative management has been proposed as a cost-efficient and low risk approach in the management of the symptoms and impairments associated with FAIS. This survey seeks to gain a better understanding of the current practice patterns being utilised for the diagnosis and management of FAIS within contemporary physiotherapy practice.

Before undertaking this survey please read the attached information sheet.

[Information sheet](Information%20sheet)

Please click the arrow at the bottom right of the screen to proceed.

**PARTICIPANT CONSENT**

This survey is available to be completed by all registered physiotherapists in Australia, Canada, Ireland, New Zealand, South Africa and the United Kingdom, who practice in the field of musculoskeletal, orthopaedic or sports physiotherapy.

By completing this survey, you acknowledge that you have read the attached information sheet, and are fully informed of the scope and details of the survey. You accept/understand that your participation is entirely voluntary and that your answers will be consolidated with others and analysed as part of a research project. You understand that the information disclosed in this survey is anonymous, will be kept confidential, and will not be accessed by anyone other than the investigators named in this research and the relevant research assistant. You understand that you have the right to withdraw from this project at any time before its completion.

Agree

Disagree  *If selects “Disagree”* *then exit from the survey, with thank you message.*

Thank you for agreeing to participate in this practitioner survey. The survey should take approximately 15 mins to complete and will be available for a period of eight weeks. Please be aware that once you have submitted the survey you will be unable to modify your responses. Any partially completed surveys will be automatically submitted at the conclusion survey period. If you experience any difficulties completing the survey, please contact us at: [FAIS-survey@otago.ac.nz](mailto:FAIS-survey@otago.ac.nz)

Please click the arrow at the bottom right of the screen to begin the survey.

**SCREENING QUESTIONS (3 QUESTIONS)**

1. Are you a registered physiotherapy practitioner with your national and/or regional registration board?

Yes  *If* ***YES****, go to Question 2*

No  *If* ***NO****, then exit from the survey, with thank you message.*

1. In which country do you primarily work as a registered physiotherapist? If you work in more than one country, please select the country where you work most of the time.

Australia

Canada

Ireland

New Zealand

South Africa

United Kingdom

Other, please specify ___________________________  *If selects “Other” then exit from the survey with thank you message.*

1. As a practising physiotherapist what percentage of your clinical practice relates to patients with hip pain?

0 10 20 30 40 50 60 70 80 90 100

Please adjust the slider to indicate the correct percentage

*If selects “0%” then exit from the survey with a thank you message.*

**SECTION 1 – DEMOGRAPHICS AND PRACTITIONER PROFILE (5 QUESTIONS)**

1. What is your gender?

Male

Female

Gender diverse, please specify ____________________

Prefer not to answer

1. Are you a physiotherapy practitioner within a primary contact clinical setting (i.e. patients have direct access to physiotherapy without prior referral)?

Yes

No

1. Where do you work? (Please select as many boxes as appropriate)

Academic institution (e.g. education)

Hospital inpatient

Hospital outpatient

Specialist/tertiary centre

Private practice

Public sector

High performance sport

Other, please specify ______________________

1. How many years of clinical physiotherapy experience do you have? (e.g. post-initial physiotherapy qualification)

Less than 5 years

5 to 10 years

11 to 15 years

16+ years

1. Are you a member of of your national physiotherapy association? (e.g. Australian Physiotherapy Association, Canadian Physiotherapy Association, Irish Society of Chartered Physiotherapists, Physiotherapy New Zealand, South African Society of Physiotherapy, Chartered Society of Physiotherapy)

Yes  *If* ***YES****, then answer the second part of this question.*

No

*Please indicate if you are a specialist, or a member of a special interest group within your national physiotherapy association. Please select as many groups as appropriate.*

Not a specialist or member of a special interest group

Musculoskeletal/manipulative therapy

Orthopaedic physiotherapy

Sports and exercise physiotherapy

Other, please specify ______________________

This survey is comprised of **two** parts: Part 1 (Diagnosis of FAIS) and Part 2 (Management of FAIS). The questions in Part 1 seek to gain an understanding of the key features that you feel are important in the diagnosis of FAIS. Please click on the arrow at the bottom right of the screen to continue onto **Part 1: Diagnosis of FAIS**.

**SECTION 2 – PHYSIOTHERAPY DIAGNOSIS (9 QUESTIONS)**

1. How confident are you in diagnosing FAIS?

| Not confident at all | Slightly confident | Moderately confident | Very confident | Extremely confident |
| --- | --- | --- | --- | --- |
|  |  |  |  |  |

1. Do you utilise patient-reported signs/symptoms when considering a diagnosis of FAIS?

| Never | Rarely | Sometimes | Often | Always |
| --- | --- | --- | --- | --- |
|  |  |  |  |  |

If selects **Never** then progress to Question 8.

If selects any option other than **Never**: *Please indicate which patient-reported signs/symptoms you consider important.*

|  | Never | Rarely | Sometimes | Often | Always |
| --- | --- | --- | --- | --- | --- |
| Motion-related hip pain |  |  |  |  |  |
| Position-related hip pain |  |  |  |  |  |
| Buttock pain |  |  |  |  |  |
| Anterior hip/thigh pain |  |  |  |  |  |
| Groin pain |  |  |  |  |  |
| Lateral hip pain/over greater trochanter (“C sign” pain) |  |  |  |  |  |
| Mechanical symptoms (e.g. clicking, catching, locking or giving way) |  |  |  |  |  |
| Stiffness or restricted range of motion |  |  |  |  |  |
| Other, please specify _____________________________ |  |  |  |  |  |

1. Do you utilise assessments of movement/range of motion (e.g. muscle length tests, goniometry) when considering a diagnosis of FAIS?

| Never | Rarely | Sometimes | Often | Always |
| --- | --- | --- | --- | --- |
|  |  |  |  |  |

If selects **Never** then progress to Question 9.

If selects any option other than **Never**: *Please indicate which movement impairments (e.g. either painful or restricted) you consider important.*

|  | Never | Rarely | Sometimes | Often | Always |
| --- | --- | --- | --- | --- | --- |
| Hip flexion |  |  |  |  |  |
| Hip extension |  |  |  |  |  |
| Hip internal rotation |  |  |  |  |  |
| Hip external rotation |  |  |  |  |  |
| Hip abduction |  |  |  |  |  |
| Hip adduction |  |  |  |  |  |
| Other, please specify _____________________________ |  |  |  |  |  |

*What methods do you utilise to assess movement/range of motion when considering a diagnosis of FAIS?*

|  | Never | Rarely | Sometimes | Often | Always |
| --- | --- | --- | --- | --- | --- |
| Muscle length tests |  |  |  |  |  |
| Goniometry |  |  |  |  |  |
| Tape measure |  |  |  |  |  |
| Visual estimation of joint range of motion |  |  |  |  |  |
| Other, please specify _____________________________ |  |  |  |  |  |

1. Do you utilise special tests (e.g. FADIR, FABER) to rule in or rule out a diagnosis of FAIS?

| Never | Rarely | Sometimes | Often | Always |
| --- | --- | --- | --- | --- |
|  |  |  |  |  |

If selects **Never** then progress to Question 10.

If selects any option other than **Never**: *Please indicate which special tests you consider important.*

|  | Never | Rarely | Sometimes | Often | Always |
| --- | --- | --- | --- | --- | --- |
| Hip flexion adduction internal rotation (FADIR) test |  |  |  |  |  |
| Hip flexion abduction external rotation (FABER) test |  |  |  |  |  |
| Hip flexion-internal rotation test |  |  |  |  |  |
| Internal rotation-flexion-axial compression test |  |  |  |  |  |
| Hip quadrant test (or Scour test) |  |  |  |  |  |
| Fitzgerald test/labral stress test |  |  |  |  |  |
| Thomas test |  |  |  |  |  |
| Other, please specify _____________________________ |  |  |  |  |  |

1. Do you assess strength when considering a diagnosis of FAIS?

| Never | Rarely | Sometimes | Often | Always |
| --- | --- | --- | --- | --- |
|  |  |  |  |  |

If selects **Never** then progress to Question 11.

If selects any option other than **Never**: *Please indicate which strength impairments you consider important.*

|  | Never | Rarely | Sometimes | Often | Always |
| --- | --- | --- | --- | --- | --- |
| Hip flexion |  |  |  |  |  |
| Hip extension |  |  |  |  |  |
| Hip adduction |  |  |  |  |  |
| Hip abduction |  |  |  |  |  |
| Hip internal rotation |  |  |  |  |  |
| Hip external rotation |  |  |  |  |  |
| Trunk/core |  |  |  |  |  |
| Other, please specify _____________________________ |  |  |  |  |  |

*What methods do you utilise to assess strength when considering a diagnosis of FAIS?*

|  | Never | Rarely | Sometimes | Often | Always |
| --- | --- | --- | --- | --- | --- |
| Manual muscle tests (e.g. Oxford Scale) |  |  |  |  |  |
| Handheld dynamometry |  |  |  |  |  |
| Groin bar/force frame testing system |  |  |  |  |  |
| Isokinetic dynamometry |  |  |  |  |  |
| Dynamic/functional assessment (e.g. repetition maximum) |  |  |  |  |  |
| Other, please specify _____________________________ |  |  |  |  |  |

1. Do you utilise functional tests (e.g. squat, single leg squat) to inform your clinical decision making when considering a diagnosis of FAIS?

| Never | Rarely | Sometimes | Often | Always |
| --- | --- | --- | --- | --- |
|  |  |  |  |  |

If selects **Never** then progress to Question 12.

If selects any option other than **Never**: *Please indicate which functional tests you consider important.*

|  | Never | Rarely | Sometimes | Often | Always |
| --- | --- | --- | --- | --- | --- |
| Single-leg stance |  |  |  |  |  |
| Single-leg squat |  |  |  |  |  |
| Bilateral squat |  |  |  |  |  |
| Gait analysis (e.g. walking/running) |  |  |  |  |  |
| Hop or jump |  |  |  |  |  |
| Other, please specify _____________________________ |  |  |  |  |  |

1. Do you utilise balance assessment (e.g. single leg balance) to inform your clinical decision making when considering a diagnosis of FAIS?

| Never | Rarely | Sometimes | Often | Always |
| --- | --- | --- | --- | --- |
|  |  |  |  |  |

If selects **Never** then progress to Question 13.

If selects any option other than **Never**: *Please indicate which balance tests you consider important.*

|  | Never | Rarely | Sometimes | Often | Always |
| --- | --- | --- | --- | --- | --- |
| Single leg balance |  |  |  |  |  |
| Star excursion balance test (SEBT) |  |  |  |  |  |
| Other, please specify _____________________________ |  |  |  |  |  |

1. Do you utilise information from imaging to inform your clinical decision making when considering a diagnosis of FAIS?

| Never | Rarely | Sometimes | Often | Always |
| --- | --- | --- | --- | --- |
|  |  |  |  |  |

If selects any option other than **Never**: *Please indicate which types imaging you utilise to assist in your diagnosis of FAIS.*

|  | Never | Rarely | Sometimes | Often | Always |
| --- | --- | --- | --- | --- | --- |
| X-ray |  |  |  |  |  |
| Computerised tomography |  |  |  |  |  |
| Magnetic resonance imaging |  |  |  |  |  |
| Magnetic resonance arthrogram |  |  |  |  |  |
| Ultrasound |  |  |  |  |  |
| Other, please specify _____________________________ |  |  |  |  |  |

If selects **Never:** *What are your reasons for you not considering imaging when confirming a diagnosis of FAIS? Please select as many options as appropriate.*

Imaging does not provide additional information to guide diagnosis

Limited or no access to imaging

Prohibitive cost of imaging

Patients unwilling to undertake imaging

Not supported by evidence

Other, please specify ____________________________

1. Any additional comments regarding diagnosis?

The questions in Part 2 seek to gain an understanding of the key features that you consider important in the management of FAIS.

Please click on the arrow at the bottom right of the screen to continue onto **Part 2: Management of FAIS.**

**SECTION 3 – PHYSIOTHERAPY MANAGEMENT (18 QUESTIONS)**

1. Overall, how confident are you in managing patients with FAIS?

| Not confident at all | Slightly confident | Moderately confident | Very confident | Extremely confident |
| --- | --- | --- | --- | --- |
|  |  |  |  |  |

1. How many patients with FAIS do you see in an average month?

1 to 2

3 to 5

6 to 10

more than 10

1. On average how many treatment sessions would you provide for patients with FAIS (including the first assessment)?

1 to 3

4 to 10

11 to 15

16+

1. Do any of the following factors influence the number of treatments you provide patients with FAIS? Please select as many options as appropriate.

Cost of treatment

Funding limits (e.g. government or insurance)

Assess to treatment (e.g.transportation, geographical barriers)

Other, please specify _____________________________

Not applicable

1. What is the typical outcome of physiotherapy treatment for most of your patients with FAIS?

| Much worse | Worse | Somewhat worse | No change | Somewhat better | Better | Much better |
| --- | --- | --- | --- | --- | --- | --- |
|  |  |  |  |  |  |  |

1. Do you use manual therapy (e.g joint/soft tissue techniques) in your management of FAIS?

| Never | Rarely | Sometimes | Often | Always |
| --- | --- | --- | --- | --- |
|  |  |  |  |  |

If selects **Never** then progress to Question 21.

If selects any option other than **Never**: *Please indicate which type of manual therapy you use.*

|  | Never | Rarely | Sometimes | Often | Always |
| --- | --- | --- | --- | --- | --- |
| Joint mobilisation (e.g. Maitland, Kaltenborn) |  |  |  |  |  |
| Mulligan mobilisation with movement |  |  |  |  |  |
| Muscle energy techniques |  |  |  |  |  |
| Massage therapy (e.g. soft tissue massage, trigger point massage) |  |  |  |  |  |
| Other, please specify _____________________________ |  |  |  |  |  |

*If you utilise manual therapy to target joints in the management of FAIS, in which region do you most commonly apply these techniques?*

|  | Never | Rarely | Sometimes | Often | Always |
| --- | --- | --- | --- | --- | --- |
| Lumbar spine |  |  |  |  |  |
| Sacroiliac joint |  |  |  |  |  |
| Hip joint |  |  |  |  |  |
| Knee joint |  |  |  |  |  |
| Other, please specify _______________________________ |  |  |  |  |  |

*If you use soft tissue techniques in the management of FAIS, which anatomical areas do you most commonly treat?*

|  | Never | Rarely | Sometimes | Often | Always |
| --- | --- | --- | --- | --- | --- |
| Lumbar region |  |  |  |  |  |
| Gluteal region (e.g. gluteus maximus/medius) |  |  |  |  |  |
| Hip adductors |  |  |  |  |  |
| Hip flexors |  |  |  |  |  |
| Thigh (e.g. quadriceps, hamstrings) |  |  |  |  |  |
| Other, please specify _______________________________ |  |  |  |  |  |

1. Do you use exercise therapy (e.g strengthening, neuromuscular training) in your management of FAIS?

| Never | Rarely | Sometimes | Often | Always |
| --- | --- | --- | --- | --- |
|  |  |  |  |  |

If selects **Never** then progress to Question 22.

If selects any option other than **Never**: *Please indicate the method(s) of exercise therapy you utilise in your management of FAIS.*

|  | Never | Rarely | Sometimes | Often | Always |
| --- | --- | --- | --- | --- | --- |
| Strengthening |  |  |  |  |  |
| Neuromuscular control/retraining |  |  |  |  |  |
| Balance retraining |  |  |  |  |  |
| Cardiovascular |  |  |  |  |  |
| Other, please specify _______________________________ |  |  |  |  |  |

If selects any option other than **Never** with reference to strengthening in Question 21, then will be directed to the following additional questions.

*Which of the following strengthening methods do you use in your management of FAIS?*

|  | Never | Rarely | Sometimes | Often | Always |
| --- | --- | --- | --- | --- | --- |
| Isometric |  |  |  |  |  |
| Weight bearing |  |  |  |  |  |
| Non-weight bearing |  |  |  |  |  |
| Resistance (e.g. elastic bands, free-weights, pulleys etc) |  |  |  |  |  |
| Plyometrics |  |  |  |  |  |
| Other, please specify _______________________________ |  |  |  |  |  |

*Which muscles do you typically target for strengthening?*

|  | Never | Rarely | Sometimes | Often | Always |
| --- | --- | --- | --- | --- | --- |
| Hip adductors |  |  |  |  |  |
| Hip abductors (e.g. gluteus medius/minimus) |  |  |  |  |  |
| Hip flexors (e.g. tensor fascia latae, iliopsoas) |  |  |  |  |  |
| Hip extensors (e.g. gluteus maximus) |  |  |  |  |  |
| Deep hip external rotators |  |  |  |  |  |
| Thigh (e.g. quadriceps, hamstrings) |  |  |  |  |  |
| Trunk muscles (e.g. core, spine) |  |  |  |  |  |
| Other, please specify _______________________________ |  |  |  |  |  |

If selects any option other than **Never** with reference to neuromuscular control/retraining in Question 21, then will be directed to the following additional question*.*

*Which of the following approaches to neuromuscular control do you use in your management of FAIS?*

|  | Never | Rarely | Sometimes | Often | Always |
| --- | --- | --- | --- | --- | --- |
| Functional movement retraining (e.g. sitting, standing, squatting) |  |  |  |  |  |
| Ballistic movement retraining (e.g. landing control) |  |  |  |  |  |
| Gait retraining (e.g. walking, running) |  |  |  |  |  |
| Proprioceptive retraining |  |  |  |  |  |
| Other, please specify _______________________________ |  |  |  |  |  |

1. Do you target flexibility (e.g. joint range of motion and/or stretching) as part of your management of FAIS?

| Never | Rarely | Sometimes | Often | Always |
| --- | --- | --- | --- | --- |
|  |  |  |  |  |

1. Do you use electrophysical agents (e.g. electrotherapy, thermal) in your management of FAIS?

| Never | Rarely | Sometimes | Often | Always |
| --- | --- | --- | --- | --- |
|  |  |  |  |  |

1. Do you use acupuncture or dry needling in your management of FAIS?

| Never | Rarely | Sometimes | Often | Always |
| --- | --- | --- | --- | --- |
|  |  |  |  |  |

1. Do you use taping in your management of FAIS?

| Never | Rarely | Sometimes | Often | Always |
| --- | --- | --- | --- | --- |
|  |  |  |  |  |

1. Do you provide specific patient education as part of your regular physiotherapy management of FAIS?

| Never | Rarely | Sometimes | Often | Always |
| --- | --- | --- | --- | --- |
|  |  |  |  |  |

If selects **Never** then progress to Question 27.

If selects any option other than **Never**: *Please indicate which of the following areas do you provide education on.*

|  | Never | Rarely | Sometimes | Often | Always |
| --- | --- | --- | --- | --- | --- |
| Pain neuroscience |  |  |  |  |  |
| Pathoanatomical theory of FAIS |  |  |  |  |  |
| Activity modification/load management |  |  |  |  |  |
| Lifestyle modification |  |  |  |  |  |
| Other, please specify _______________________________ |  |  |  |  |  |

1. Do you use patient-reported outcome measures to assess and/or record progress when managing FAIS?

| Never | Rarely | Sometimes | Often | Always |
| --- | --- | --- | --- | --- |
|  |  |  |  |  |

If selects **Never** then progress to Question 28.

If selects any option other than **Never**: *Please indicate which patient-reported outcome measures you use as part of your management. Please select as many options as appropriate.*

Copenhagen Hip and Groin Outcome Score (HAGOS)

International hip outcome tool – 33 item (iHOT-33)/12 item (iHOT-12)

Hip outcome score (HOS)

Non-arthritic hip score (NAHS)

Modified Harris Hip Score (MHHS)

Hip disability and osteoarthritis outcome score (HOOS)

The Western Ontario and McMaster Universities Osteoarthritis Index (WOMAC)

Pain Visual Analogue Scale (VAS)/Pain Numerical Rating Scale (NRS)

Patient specific functional score (PSFS)

Global Rating of Change (GROC) scale

Other, please specify _______________________________

**Non-physiotherapy management and referral**

1. Please indicate if would you would consider non-physiotherapy treatment options (e.g. intra-articular injections, surgical interventions) in the management of FAIS.

| Never | Rarely | Sometimes | Often | Always |
| --- | --- | --- | --- | --- |
|  |  |  |  |  |

If selects **Never** then progress to Question 31.

If selects any option other than **Never:** *What are your criteria for onward referral for non-physiotherapy treatment options. Please select as many options as appropriate.*

Patient not responding to treatment after a specified number of sessions  Please specify the number of sessions _______

Conclusion of funded treatment sessions

At the patient’s request

Specific assessment findings, please specify ____________________________

Specific imaging findings, please specify ____________________________

Other, please specify ____________________________

Not applicable

1. Do you consider referral for intra-articular hip injections in the management of patients with FAIS?

| Never | Rarely | Sometimes | Often | Always |
| --- | --- | --- | --- | --- |
|  |  |  |  |  |

If selects **Never** then progress to Question 30.

If selects any option other than **Never:** *Please indicate which method of intra-articular hip injection is utilised when patients with FAIS under your care are referred for therapeutic injections. Please select as many options as appropriate.*

Corticosteroid injections (CSI)

Hylaronic acid injections

Platelet-rich plasma (PRP) injections

Other, please specify _______________________________

Not sure

*What are your beliefs regarding the use of intra-articular injections in the management of FAIS? Please select as many options as appropriate.*

Reduces pain

Reduces inflammation

Aids in healing associated pathology

Provides a window of opportunity initiate active rehabilitation

Improves functional capacity

Is useful as a stand-alone treatment

Should always be provided in conjunction with an active rehabilitation (e.g. exercise)

Has a positive effect on joint health (e.g. articular pathology)

Has no negative effects

Other, please specify _______________________________

1. Do you consider referral for surgical interventions in the management of patients with FAIS?

| Never | Rarely | Sometimes | Often | Always |
| --- | --- | --- | --- | --- |
|  |  |  |  |  |

If selects **Never** then progress to Question 31.

If selects any option other than **Never:** *What are your beliefs regarding surgical management of FAIS?* *Please select as many as appropriate.*

Reduces pain

Aids in healing associated pathology

Provides a window of opportunity initiate active rehabilitation

Improves functional capacity

Is useful as a stand-alone treatment

Should be combined with active rehabilitation (e.g. exercise)

Has a positive effect on joint health (e.g. articular pathology)

Has no negative effects

Other, please specify _______________________________

**Return to activity**

1. What clinical tools do you utilise to determine readiness for return to sport, work or other meaningful tasks. Please select as many as appropriate.

Patient reported outcome measures.

Functional tests/screening (e.g. performance of patients meaningful task)

Pain-free range of motion

Lower limb strength equivalent to the contralateral limb

Completion of a patient specific movement battery

Other, please specify _______________________________

1. Any additional comments regarding treatment?

**EXIT MESSAGE – MAIN SURVEY**

Thank you for taking the time to complete this survey. We greatly appreciate your contribution to this project and value your input. Your responses to the survey have been recorded and will remain anonymous.

If you have any additional queries regarding the study please do not hesitate to contact us at: [FAIS-survey@otago.ac.nz](mailto:FAIS-survey@otago.ac.nz)
